# Quenching Photo‐Induced Free Radicals Endows Superomniphobic Atmospheric Water Harvesting Hydrogels With UV Resistance for Durable Bifacial Photovoltaic Cooling

**DOI:** 10.1002/advs.78353

**Published:** 2026-10-22

**Authors:** Xiaoqi Zhou, Chengyu Yang, Rongrong Ma, Kun Zhang, Liyin Liu, Bo Wu, Renyuan Li

**Affiliations:** ^1^ School of Marine Sciences Ningbo University Ningbo People's Republic of China; ^2^ Beijing National Laboratory for Molecular Sciences Key Laboratory of Molecular Nanostructure and Nanotechnology Institute of Chemistry Chinese Academy of Sciences Beijing People's Republic of China; ^3^ School of Environmental Science and Technology Key Laboratory of Industrial Ecology and Environmental Engineering (Ministry of Education) Dalian University of Technology Dalian University of Technology People's Republic of China; ^4^ University of Chinese Academy of Sciences Beijing People's Republic of China; ^5^ Center For Carbon Neutral Chemistry Institute of Chemistry Chinese Academy of Sciences Beijing People's Republic of China

**Keywords:** bifacial PV panel cooling, functionalized fullerene C_60_, hygroscopic hydrogels, sorption‐based evaporative cooling, UV resistance

## Abstract

Bifacial photovoltaic (PV) modules constitute 75% of global PV manufacturing output and are playing an increasingly vital role in the clean energy transition. However, their operational efficiency is severely compromised by heat accumulation and dust soiling. Moreover, the rear‐side optical transparency required for bifacial light harvesting renders conventional thermal management techniques infeasible. This work reports a zero‐energy‐demand and liquid water‐free cooling strategy for bifacial PV panels: Functionalized fullerene C_60_ is homogeneously incorporated into the atmospheric water harvesting (AWH) hydrogel and terminates light‐induced radical chain reactions, enhancing the hydrogel's UV stability by over 385%. Meanwhile, the modified hydrogel surface imparts superomniphobicity with oil and water contact angles exceeding 160°, resists dust adhesion, and maintains 92.5–96.0% transmittance in the PV response spectrum. The hydrogel cooling layer has a calculated unit cost as low as $9.0 m^−2^ and delivers a daytime cooling power of 550–650 W m^−2^ when the PV panels are in operation, reducing the panel temperature by up to 14.3°C and boosting both the maximum power output and conversion efficiency by 9.03%. This strategy holds a global annual carbon mitigation potential of 537.56 million metric tons of CO_2_, offering a sustainable route for photovoltaic thermal management.

## Graphical abstract

A hygroscopic hydrogel layer functionalized with fullerene C_60_ tackles heat and dust challenges in bifacial photovoltaics (PV) panels. It resists soiling and photo‐aging, maintains high transparency, absorbs water vapor from the air during the night, and delivers strong evaporative cooling power during daytime, significantly lowering PV panel temperature and boosting PV power output.

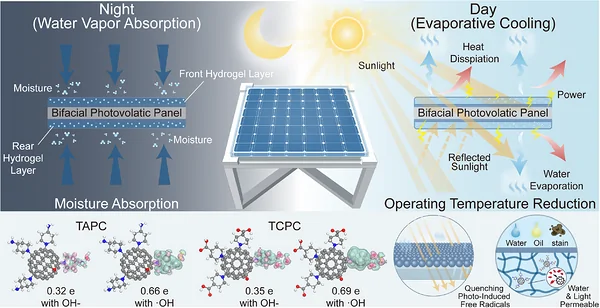

## 1 Introduction

Energy has long been considered the key driving force for the development of modern society. Electricity is one of the most important forms of energy. In 2024, the global growth rate of electricity demand reached as high as 4.4%, and it is projected that the average annual growth rate of electricity demand will remain at 3.6% between 2026 and 2030 [1]. The rapid growth of electricity demand exacerbates both energy and environmental crisis in the global scale, and in the context of carbon neutrality, the global energy system is rapidly transitioning toward cleaner and low‐carbon directions [2, 3]. As the most widely distributed renewable primary energy source on Earth, solar energy offers inherent advantages of virtually unlimited resources, independence from geographical conditions, and clean, non‐polluting nature [4]. The total amount of solar energy received by the Earth per hour reaches as high as 173 000 TWh [5], which is close to the projected total global primary energy consumption for the entire year of 2025 (180 000 TWh). Photovoltaic (PV) technology utilizes semiconductor materials to capture photons within specific wavelength ranges of incident sunlight, thereby exciting electrons from the valence band to the conduction band and forming electron‐hole pairs [6]. These pairs are subsequently separated by the internal electric field, generating an electrical potential difference and directly converting solar energy into high‐grade electrical energy, which effectively broadens its application forms and scopes [7]. In 2024, global PV power generation exceeded 2 100 TWh [8], exhibiting a rapid growth trend, and is projected to surpass wind power and hydropower to become the largest source of renewable electricity by 2029 [9]. Commercial PV modules are currently classified into monofacial and bifacial types, with bifacial modules accounting for over 75% of global manufacturing output [10]. The rear side of bifacial modules can capture reflected sunlight from the environment, allowing them to generate approximately 10% additional electricity [11].

However, two long‐standing obstacles in the PV industry severely hinder the applicability of bifacial modules while simultaneously adding to the sector's overall carbon footprint. The first is high temperature. Due to the Shockley‐Queisser limit, the theoretical maximum solar energy conversion efficiency of single‐junction silicon‐based solar cells is only 33% [12]. In practice, over 75% of incident solar energy is converted into heat, raising the operating temperature of PV panels to more than 40 °C above ambient conditions [13]. This heating effect is even more severe in bifacial modules, since both sides receive sunlight and contribute to thermal buildup. High temperatures reduce the semiconductor bandgap and intensify carrier recombination, thereby significantly degrading the power conversion efficiency (PCE) by up to 0.5% /°C [14, 15, 16]. In addition, elevated temperatures accelerate the aging of PV modules and cells, thereby compromising both system stability and shortening the lifespan [17, 18]. The second is dust accumulation. The bifacial PV modules are typically encapsulated with tempered glass, which accumulates electric charge and interacts with charged airborne particles through electrostatic forces, leading to dust attraction and deposition [19, 20]. Furthermore, most PV modules are deployed in arid and semi‐arid regions characterized by frequent dust storms and low rainfall, which further aggravate soiling on PV modules, blocking the light transmittance and thereby diminishing power generation capacity [21, 22, 23, 24]. Constrained by these challenges, the power output of commercially available bifacial PV modules under real‐world outdoor operating conditions is typically less than half of their rated value.

Water spraying is currently the most widely used PV cooling and cleaning method, leveraging the high heat capacity of water to dissipate heat and simultaneously maintaining the cleanness of PV surfaces [25, 26]. Nevertheless, such approaches suffer from several drawbacks, including system complexity, high initial capital costs, additional electricity consumption, etc [27]. Most importantly, water‐based active cooling and cleaning techniques inherently depend on a steady supply of clean freshwater. Consequently, such technology is hard to adapt in arid and semi‐arid regions where PV modules are commonly installed from economic and ecological considerations [28, 29]. To address this, a monofacial PV panel cooling strategy based on the sorption and desorption cycles of atmospheric water was proposed by Li and Wang et al. [30]. This strategy utilized a hygroscopic hydrogel for water vapor sorption and storage from the ambient air during nighttime. During daytime, the stored water is evaporated to dissipate heat from PV modules via the latent heat of evaporation to achieve a zero‐electricity consumption cooling. While the hygroscopic hydrogels are composed of complex compositions, exposure of the hydrogel to sunlight generates reactive free radicals, triggering a photo‐oxidative chain reaction [31]. It should be noted that PAM degradation in environmental systems is a complicated process, involving radical‐mediated chain scission, where impurities such as dissolved oxygen and metal ions can contribute to hydroxyl radical formation through both direct and indirect pathways [32]. This leads to photo‐induced aging of the hydrogel, significantly reducing its hygroscopicity and mechanical strength upon utilization. Furthermore, the hydrogel surface contains abundant polar hydrophilic functional groups such as amino and hydroxyl groups, which impart strong adhesiveness. As a result, the surface becomes highly susceptible to dust soiling and thus difficult to clean, which blocks water vapor pathways and reduces the sorption capacity.

Fullerene C_60_ is a spherical molecule composed of 60 carbon atoms, featuring a large conjugated π‐bond system on its surface [33]. The highly delocalized electron cloud endows them with a pronounced electron‐deficient characteristic, enabling them to act as efficient electron acceptors that capture and neutralize unpaired electrons of free radicals, thereby quenching their reactivity. During this process, fullerenes exert antioxidant activity by catalyzing the quenching of free radicals while themselves are not being consumed [34, 35]. This study is based on the functionalization of fullerene C_60_ to enable its stable dispersion within a hydrogel network through chemical modification of the fullerene carbon cage with functional groups (e.g., ─OH, ─COOH) that establish bonds with water via hydrophilic functional adducts [36], forming a hybrid system that effectively inhibits photo‐generated radical formation, terminates photo‐oxidative chain reactions, and enhances the UV resistance of the hygroscopic hydrogels [37]. Furthermore, alkylated hydrophobic silica nanospheres are employed as a coating layer for the hydrogel surface modification. Without compromising water vapor permeability or light transmittance, this coating effectively reduces surface energy and covers polar hydrophilic functional groups, ultimately imparting superomniphobic properties that effectively suppress soiling and dust adhesion of the hydrogel. The abundant Si─O─Si bonds generate phonon‐polariton resonances [38] and therefore exhibit excellent radiative cooling performance within the atmospheric infrared window, further enhancing the cooling capability. Experimental data show that the hygroscopic hydrogel exhibits a water vapor sorption capacity of up to 2.08 g g^−1^ at a relative humidity (RH) of 90%, a transparency exceeding nearly 93% within the core response spectral range of photovoltaics, a UV light stability enhancement of over 385%, and both oil and water contact angles of >160°. PV panel cooling experiments demonstrate that an averaged cooling power of approximately 550–650 W m^−2^ can be achieved under 1.2 kW m^−2^ (i.e., 1.2 Sun) of solar irradiation, leading to a maximum temperature reduction of 14.3 °C, and improvements of 9.03% in both maximum power output and conversion efficiency. Carbon emission reduction analysis indicates that this technology has a global annual carbon mitigation potential of 537.56 million metric tons (Mts). In summary, this study provides a new avenue for sustainable photovoltaic cooling, efficiency enhancement, and anti‐dust deposition protection, simultaneously achieving effective thermal management and fouling resistance for PV modules. These findings are of great significance for improving PV power generation capacity, ensuring operational stability, extending system service life, and reducing the carbon footprint of photovoltaic power generation.

## 2 Results and Discussions

This work enhances the ultraviolet resistance of hygroscopic hydrogel polymer networks by introducing two types of functionalized fullerenes with highly electron‐deficient characteristics, which catalytically quench photoinduced radicals through radical‐radical recombination, thereby terminating photooxidative chain reactions. Concurrently, the fullerenes serve as polymerization regulators, preventing aggregation through strong interactions with the polymer backbone to maintain hydrogel transparency, while significantly extending the service life of the hydrogel under direct sunlight exposure. Furthermore, a superomniphobic structure is constructed on the hydrogel surface through synergistic effects of micro‐replicated rough morphology and low surface energy modification. Such a design effectively resolves the issues of dust soiling and the rain‐leaching of hygroscopic components, which otherwise reduce optical transparency and hygroscopicity. The strategy proposed in this work effectively overcomes the bottlenecks of light‐induced aging and surface fouling in hygroscopic hydrogels, rendering polymer‐based hygroscopic hydrogels promising for the cooling and performance enhancement of high‐efficiency, bifacial PV panels with zero water and zero electricity consumption.

### 2.1 Synthesis of PAM‐A_x_/C_x_‐CaCl_2_@S Hydrogel and Device Assembly

The PAM‐ A_x_/C_x_ ‐CaCl_2_@S hydrogel was synthesized through a 2‐step process (Scheme 1). The procedure involved dispersing acrylamide monomer in a 0.4 g mL^−1^ CaCl_2_ solution, and subsequently adding amino/carboxylated fullerene, followed by polymerization using MBAA as crosslinker, KPS as initiator, and TEMED as catalyst. The resulting fullerene‐embedded hydrogel was denoted as PAM‐ Ax/Cx ‐CaCl_2_, and then functionalized by coating hydrophobic silica particles, endowed with superomniphobic properties while maintaining accessibility of water vapor and optical transparency. Finally, the functionalized samples, denoted as PAM‐ Ax/Cx ‐CaCl_2_@S hydrogel, were adhered to the glass encapsulation of a bifacial PV panel, forming a protective cooling layer. This structured panel was subsequently placed within a custom‐designed, biodegradable polylactic acid (PLA) framework, thus completing the integrated bifacial PV panel cooling system. Preliminary screening was conducted based on both UV‐resistance testing and transparency evaluation to confirm the TAPC (tetra[4‐(amino)piperidin‐1‐yl]C_60_ epoxide) and TCPC (tetra[4‐(carboxyl)piperidin‐1‐yl]C_60_ epoxide) concentrations in the hygroscopic hydrogel in this work (Note S1), which were determined to be 0.01 and 0.03 mg mL^−1^ for TAPC and TCPC, respectively. Therefore, the hydrogel samples used for performance evaluation were denoted as PAM‐A1‐CaCl_2_, PAM‐C3‐CaCl_2_ for uncoated samples, and PAM‐A1‐CaCl_2_@S, PAM‐C3‐CaCl_2_@S for hydrophobic SiO_2_‐coated samples.

**SCHEME 1:**
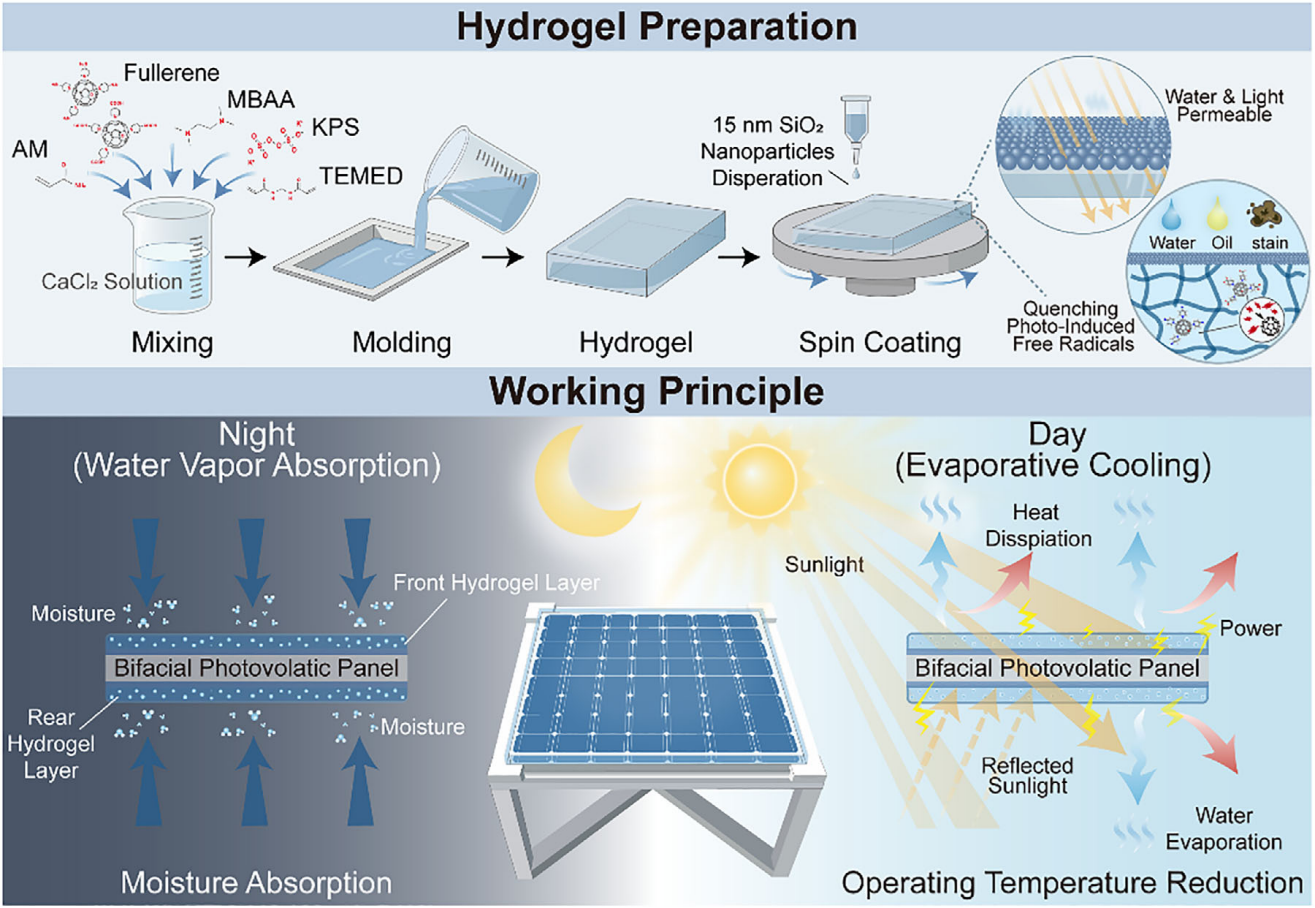
Schematic of material preparation and functionalization process.

Scanning electron microscopy (SEM) images revealed the porous structures of the hydrogels, which served as channels for efficient water vapor transport (Figures S4a and S7a). Hydrophobic silica spheres (SiO_2_) with a diameter of 15 nm were used to construct roughness structures with low surface energy on the hydrogel surface. As seen in Figure 1a, two distinct peaks at 830 and 690 eV, corresponding to F KLL and F 1s, respectively, can be observed for the modified SiO_2_ compared to the unmodified SiO_2_, indicating that the PFOES modification was successful. Along with the F KLL and F 1s peaks, Si 2s and Si 2p peaks can also be observed at 154 and 103.5 eV, respectively, for PAM‐A1@S and PAM‐C3@S when compared to the PAM‐A1 and PAM‐C3 samples, confirming that the coating was successful. In contrast to the smooth microscopic morphology of the original hydrogels, the surface of the modified hydrogels coated with hydrophobic silica particles exhibited a loosely stacked coating layer. This layer provided the microstructures required for superomniphobic modification and accessible routes for water molecule transportation across the hydrogel‐ambient air interface (Figures S4f and S7f). X‐ray diffraction (XRD) patterns also confirmed the existence of the SiO_2_ coating layer (Figure S11), as seen in Figure 1b; three characteristic peaks at 2θ of 28.8°, 47.0°, and 47.9° corresponded to (111), (220), and (022) planes of SiO_2_, respectively, and can be observed in PAM‐A1@S and PAM‐C3@S while absent in the uncoated PAM‐A1 and PAM‐C3 samples.

**FIGURE 1:**
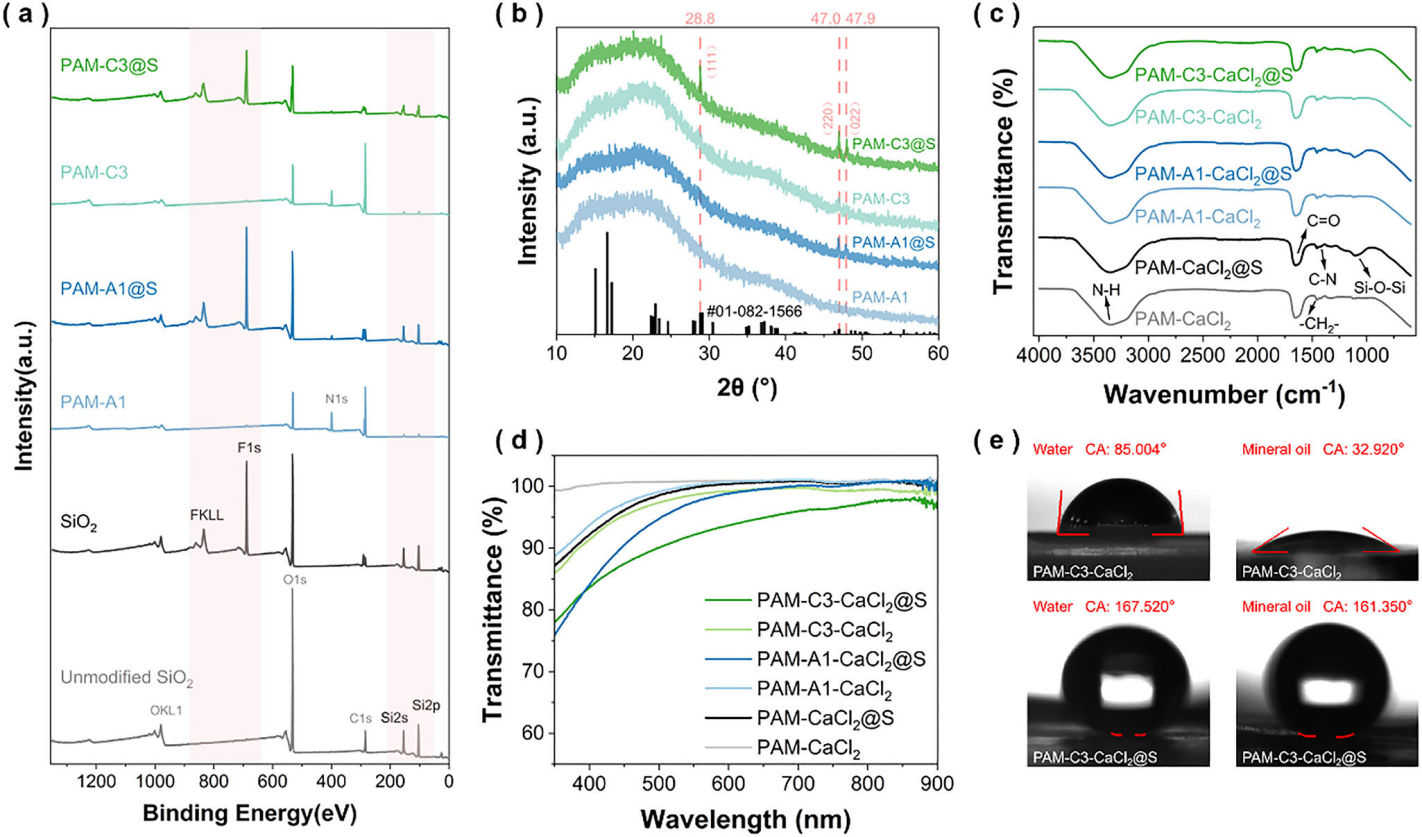
Material properties of the hydrogel. (a) XPS survey spectra of unmodified SiO_2_, hydrophobic SiO_2_, fullerene‐embedded hydrogels, and coated hydrogels. (b) XRD patterns of coated and uncoated hydrogels. (c) FTIR spectra and (d) UV–vis spectra of the samples. (e) Water and mineral oil contact angles of PAM‐CaCl_2_‐C3 hydrogel and PAM‐CaCl_2_‐C3@S hydrogel.

Further analysis utilizing Fourier Transform Infrared Spectroscopy (FTIR) provided insights into both the functional group composition and the structural integrity of the hydrogel before and after coating (Figure 1c). The broad peak at 3365 cm^−1^ primarily corresponds to the N‐H stretching vibration of the PAM amide groups, possibly overlapped by a minor contribution from O‐H stretching [39]. The peak at ∼1640 cm^−1^ mainly represents the C═O stretching vibration in the amide I band of PAM [40, 41], the peaks at 1453 and 1411 cm^−1^ originate from the bending vibration of ─CH_2_‐ and the C─N stretching vibration in the amide group, respectively, which are typical characteristics of the PAM backbone [42]. No significant shifts in the peak positions before and after coating were observed, indicating that the coating did not disrupt the main structure of the PAM hydrogel; conversely, the appearance of the asymmetric stretching vibration of the Si─O─Si network at ∼1097 cm^−1^ confirmed the deposition of the SiO_2_ layer onto the surface of the hydrogels [43]. The transmittance of the hydrogels was evaluated using a UV–vis spectrophotometer within the wavelength range of 300–900 nm (Figure 1d). As seen, on the one hand, the embedment of fullerene reduces the optical transmittance of the hydrogels within the UV range (i.e., 300–380 nm), which is favorable for reducing the aging of solar cell under sunlight irradiation. On the other hand, light transmittance of PAM‐CaCl_2_, PAM‐CaCl_2_@S, PAM‐A1‐CaCl_2_, PAM‐A1‐CaCl_2_@S, PAM‐C3‐CaCl_2_, and PAM‐C3‐CaCl_2_@S within the PV responsive spectrum of the solar cell was measured and integrated to be 100.0%, 98.6%, 99.2%, 96.0%, 97.5%, and 92.5%, respectively, demonstrating limited optical transparency loss due to fullerene embedment and hydrophobic coating layer. Liquid contact angle (CA) at the surface of the hydrogel before and after surface modification was measured to be 85.0° and 167.5° for water, and 32.9° and 161.4° (Figure 1e) for mineral oil, demonstrating the superomniphobicity of the coated hydrogel surface. The optical transparency and the superomniphobic wetting behavior collectively confirmed the great applicability potential of PAM‐ A1/C3 ‐CaCl_2_@S hydrogels to be used for bifacial PV panel cooling.

### 2.2 UV Resistance and Water Vapor Sorption Behavior of PAM‐ Ax/Cx ‐CaCl_2_@S Hydrogel

To evaluate the UV resistance of fullerene‐embedded hydrogels, a high‐dosage UV exposure test was conducted. Hydrogel samples were placed in a UV aging chamber and exposed under a 50 W 365 nm UV lamp for 3 h, equivalent to an exposure dosage of 1.26 × 10^6^ J m^−2^. Subsequently, the mechanical properties of the hydrogels before and after UV exposure were evaluated using a universal testing machine. After 3 h of UV irradiation, the PAM‐CaCl_2_ hydrogel exhibited a drastic decline in both elongation at break (from approximately 5 900% to 4 100%) and tensile strength (from 0.19 to 0.09 MPa). In contrast, PAM‐A1‐CaCl_2_ and PAM‐C3‐CaCl_2_ maintained high elongation at break values of 9 500% and 6 900%, respectively, and tensile strengths of 0.15 and 0.18 MPa, respectively, demonstrating their outstanding UV resistance (Figure 2a). Electron spin resonance (ESR) test detected strong characteristic peaks of hydroxyl free radicals in the PAM‐CaCl_2_ hydrogel under direct UV exposure, while this typical signal cannot be observed in the PAM‐A1‐CaCl_2_ and PAM‐C3‐CaCl_2_ samples. These results demonstrated that even trace amounts of TAPC and TCPC can achieve highly efficient quenching of photo‐induced free radicals, thereby explaining the outstanding UV aging resistance of the modified hydrogels observed in the mechanical tests (Figure 2b).

**FIGURE 2:**
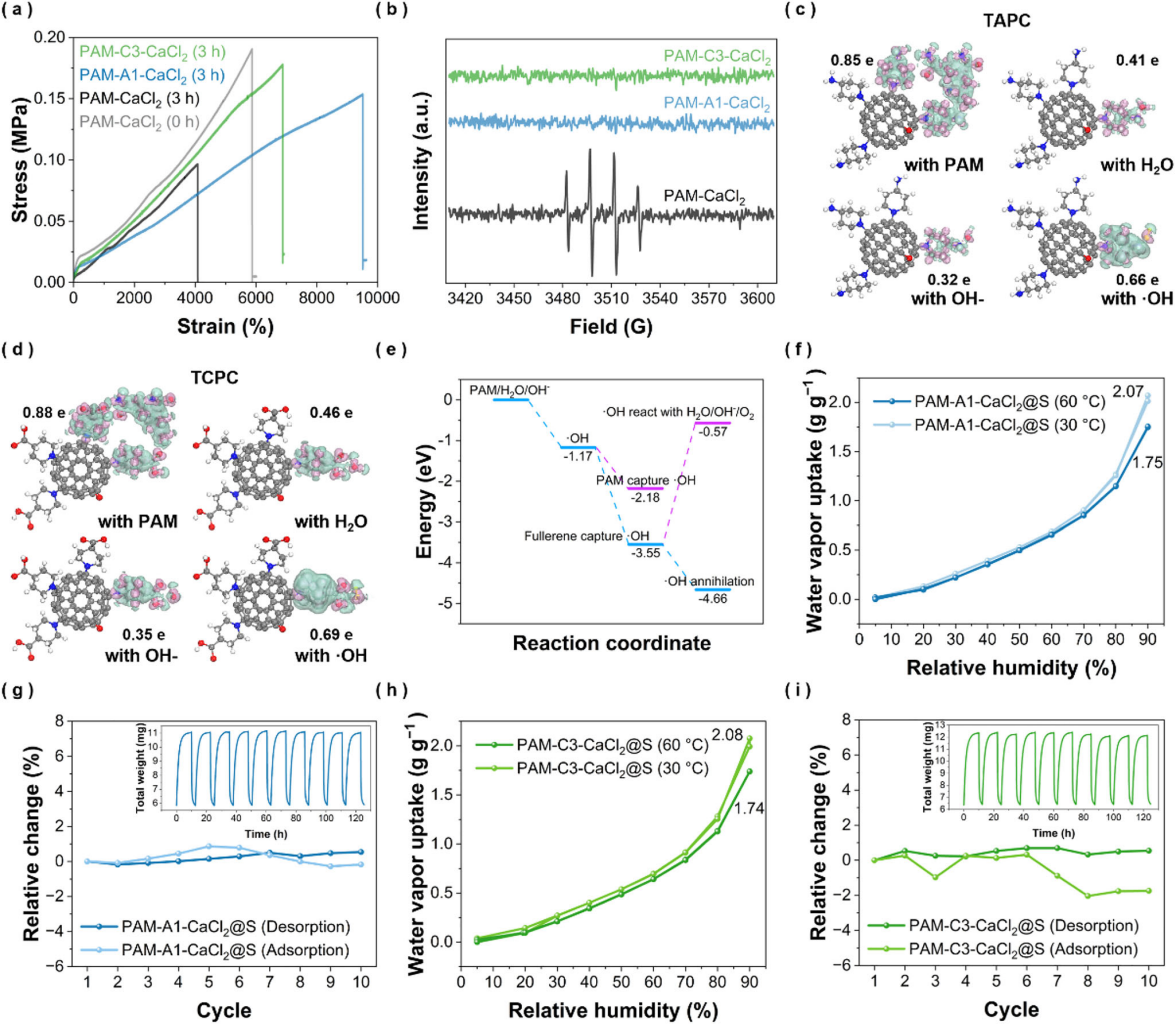
UV resistance and water vapor sorption behavior of PAM‐ Ax/Cx ‐CaCl_2_@S hydrogel. (a) Strain–stress curves of the hydrogel samples before and after high‐dosage UV exposure. (b) Electron spin resonance (ESR) spectrum of PAM hydrogel and PAM‐A1/C3 hydrogel upon UV exposure. Molecular dynamics (MD) simulation results of (c) charge distribution of TAPC and (d) TCPC at different states, (e) reaction energy barrier. Water vapor sorption/desorption isotherms and cycling stability tests of PAM‐A1‐CaCl_2_@S hydrogel (f), (g) and PAM‐C3‐CaCl_2_@S hydrogel (h), (i), respectively.

A mechanistic study was carried out by density functional theory (DFT) and molecular dynamics (MD) calculations (Note S2). The calculations demonstrated that TAPC and TCPC can be firmly anchored within the PAM network, forming stable radical‐responsive sites, and also revealing the highest interaction energies with ·OH (Figures S21 and S22). Charge distribution and charge transfer calculations evidencing that the charge transfer between fullerenes and ·OH is significantly higher than that with H_2_O and OH^−^. This is consistent with the well‐established role of the highly electron‐deficient π‐conjugated fullerene cage as an efficient electron acceptor for radical quenching through single‐electron transfer [34, 44, 45]. For both TAPC and TCPC, more prominent and localized regions of charge accumulation and depletion are observed at the interface upon interaction with ·OH. The charge density is mainly distributed between the π‐electron system of the fullerene surface and the adjacent radical, indicating strong electronic coupling at the interface. For TAPC, the charge transfer values with PAM, H_2_O, OH^−^, and ·OH are approximately 0.85, 0.41, 0.32, and 0.66 e, respectively, while for TCPC, the corresponding values are 0.88, 0.46, 0.35, and 0.69 e, suggesting that fullerenes can act as electron‐buffering and delocalization platforms through their π‐conjugated framework (Figure 2c,d).

MD simulations demonstrate that once ·OH is present in the PAM/H_2_O/CaCl_2_ system, the fullerene exhibits a much stronger thermodynamic driving force for ·OH capture than PAM (Figure S23, The colored spheres represent different atoms and radical species: gray for C, red for O, white for H, blue for N, and yellow for ·OH). While PAM is capable of interacting with ·OH, leading to a free energy decrease to approximately −2.20 eV, the capture of ·OH by fullerene results in a significantly lower free energy of ‐3.55 eV. This substantial energy difference demonstrates that fullerene exhibits a much stronger thermodynamic driving force for radical capture and stabilization compared to PAM (Figure 2e). The free energy decreases further from −3.55 eV (fullerene‐·OH complex) to −4.66 eV during the radical annihilation step, suggesting that the quenching process is highly favorable. In contrast, if ·OH continues to react with surrounding H_2_O, OH^−^, or O_2_, the corresponding free energy only reaches approximately −0.57 eV, which is significantly higher than that of the fullerene‐mediated pathway.

The frontier molecular orbital (HOMO/LUMO) analysis was employed to clarify the hydroxyl radical characteristics in the hydrogel system (Figure S24). The LUMO energies of TAPC and TCPC are calculated to be −6.117 and −6.358 eV, respectively, both lower than the HOMO level of ·OH (−6.023 eV), enabling strong interfacial orbital coupling and radical capture. The energy gap between the HOMO of ·OH (−6.023 eV) and the LUMO of fullerenes (−6.117 to −6.358 eV) is extremely small (∼0.1–0.3 eV), providing near‐resonant conditions for efficient frontier orbital coupling and charge transfer. In contrast, the LUMO levels of H_2_O (−1.877 eV) and OH─ (−3.124 eV) are significantly higher, making them less competitive in interacting with ·OH. Therefore, once ·OH is generated, it is thermodynamically and electronically more favorable to interact with fullerene rather than undergo further reactions with other species in the system, making the embedment of TAPC and TCPC capable of cutting the chain reaction for photo‐induced polymer aging.

The water vapor sorption/desorption properties of the TAPC/TCPC embedded hygroscopic hydrogels were analyzed by a dynamic vapor sorption (DVS) analyzer under constant temperatures of 30°C and 60°C at varied relative humidities (RH, %). For both PAM‐A1‐CaCl_2_@S and PAM‐C3‐CaCl_2_@S, their water vapor uptake behaviors are almost identical; water vapor uptake capacities increased with increasing RH, reaching a maximum of ∼2.08 g g^−1^ at RH 90%, 30°C and ∼ 1.75 g g^−1^ at RH 90%, 60°C, respectively. When the relative humidity was at a low level, for example, at RH 40%, the water vapor uptake was ∼0.4 g g^−1^. The desorption curves almost completely overlap with the sorption curves, indicating reversible sorption/desorption behavior driven by the water vapor pressure gradient (Figure 2f,h). The cycling tests further demonstrate highly stable and reliable water vapor sorption‐desorption performance for both hydrogels, which is critical for practical applications. After 10 cycles, only a negligible decline in water vapor uptake was observed. The same sample (PAM‐A1‐CaCl_2_@S) was further subjected to extended cycling after 11 months of unprotected storage under laboratory conditions. In one word, even after 40 cycles, the overall sorption‐desorption fluctuation remained within approximately 2% (Figure 2g,i and Figure S17).

The determination of long‐term salt retention capability of the hydrogel was further carried out. Briefly, a piece of hydrogel with dimensions of 10 × 10 × 3 mm was placed in a constant temperature and humidity chamber (25°C, 90% RH) for 2 h for water vapor sorption and subsequently transferred to a box oven for dehydration at 90°C for 1 h, which was counted as one manual sorption‐desorption cycle. Before the cycling test, the Ca^2+^ content measured by inductively coupled plasma‐optical emission spectrometry (ICP‐OES) was approximately 7.74 wt.%, and after 25 cycles it decreased slightly to approximately 7.72 wt.%, corresponding to a reduction of only about 0.3%. For Cl^−^, the concentration measured by ion chromatography (IC) was 109.13 mg L^−1^ before cycling and 106.28 mg L^−1^ after 25 cycles, with a maximum relative change of approximately 3.5% (Figure S16). These results provided direct evidence of material stability even under conditions that were much more severe than the practical PV cooling conditions.

### 2.3 Bifacial PV Panel Cooling Test in Laboratory Conditions

The bifacial PV cooling performance was first evaluated under simulated sunlight in controlled laboratory conditions to investigate the effectiveness of the hydrogel cooling layer. Batch experiments were conducted to assess two key parameters influencing the cooling performance: (1) the thickness of the hydrogel, which affects the maximum water vapor uptake capacity and the amount of water available for evaporative cooling of the bifacial PV panels, and (2) the configuration of the hydrogel‐PV assembly, including attachment of the hydrogel to the front side, the rear side, or both sides of the PV panel (Note S3). The Parameter determination tests indicated that the hydrogel thickness of 3 mm and the attachment of a hygroscopic hydrogel cooling layer to both sides of the bifacial PV panels (denoted as PAM‐A1‐CaCl_2_@S‐B or PAM‐C3‐CaCl_2_@S‐B) represent the optimal conditions; therefore, these parameters were adopted in the subsequent performance evaluations.

The in‐lab bifacial PV panel cooling test results of PAM‐C3‐CaCl_2_@S‐B and PAM‐A1‐CaCl_2_@S‐B are presented in Figure 3 and Figure S25. Since both samples exhibit similar test performance, the following analysis and discussions are based on PAM‐C3‐CaCl_2_@S‐B as a representative example. The temperature profiles of the bifacial PV panels are depicted in Figure 3a–c, and Note S4. As seen, the bifacial PV panel integrated with the PAM‐C3‐CaCl_2_@S cooling layer reached an equilibrium temperature 6–12 °C lower than the pristine panel under solar irradiances of 0.8, 1.0, and 1.2 kW m^−2^. The sharp contrast observed in the IR images captured at 0.5 h time intervals further confirmed the temperature differences resulting from evaporative cooling throughout the test (Figure 3d). The performance indicators of the PV panel, including open‐circuit voltage (V_oc_), maximum power output (P_max)_, fill factor (FF), and efficiency, were calculated and compared based on the current‐voltage (I‐V) curves obtained during the test (Figure 3e,f and Figures S31 and S32). As can be seen, for both cases with and without the cooling layer, as the irradiation time increased, the V_oc_, P_max_, FF, and efficiency data demonstrated a rapid decline within the first 10 min, followed by a gradual slowdown and eventual stabilization. The cooling layer effectively delayed the performance degradation at this initial stage and improved all the performance indicators at the stable outputs. For quantitative assessment, two parameters are defined in this work: the ***performance degradation delaying ratio*** (PDDR, Equation 1), quantifying the mitigation effect of the integrated hygroscopic cooling layer on PV performance degradation induced by temperature shifts from the initial to the operating state; and the ***performance enhancement ratio*** (PER, Equation 2), characterizing the improvement in steady‐state outputs at equilibrium.

**FIGURE 3:**
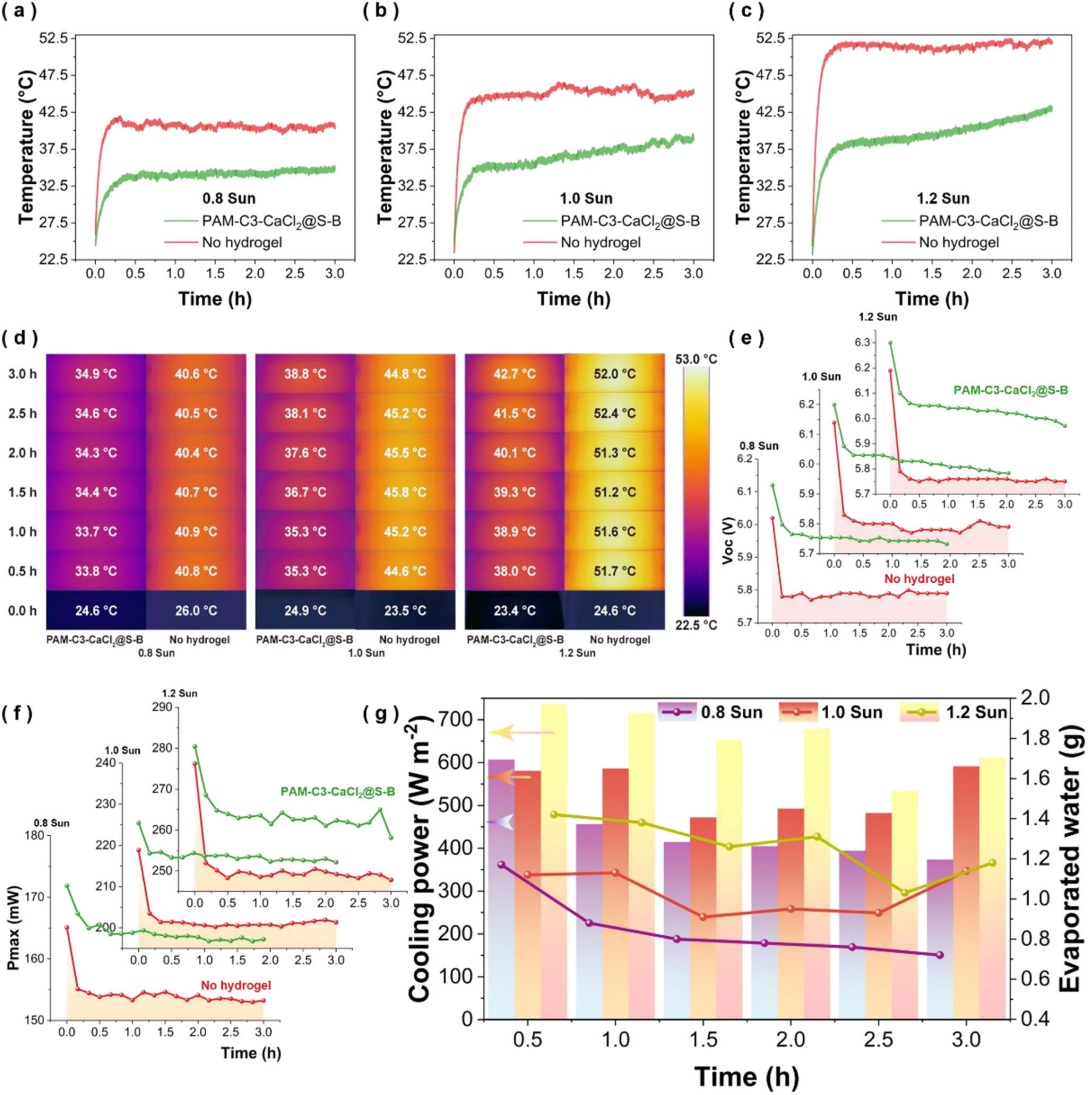
Bifacial PV panel cooling results under laboratory conditions. (a–c) Temperature profiles of the bifacial PV panels with and without the PAM‐C3‐CaCl_2_@S cooling layer. (d) IR images of the bifacial PV panel under different solar irradiance. (e) V_oc_ and (f) P_max_ of the bifacial PV panel with or without cooling. (g) Cooling power (columns) and hourly weight‐loss profiles (curves) of the hydrogel during the test.

(1) $$PDDR=\frac{\left(PV_{ini}-PV_{10}\right)-\left(CPV_{ini}-CPV_{10}\right)}{PV_{ini}-PV_{10}}\;\times 100\%$$

where *PV_ini_* and *PV*_10_ are the performance indicator data at the initial state and at 10 min of a pristine bifacial PV panel without cooling, respectively, *CPV_ini_* and *CPV*_10_ are the performance indicator data at the initial state and at 10 min of the bifacial PV panel with a cooling layer, respectively. The performance degradation in the first 10 min can be found in Table S1, and the calculated PDDR can be found in Table S2. Briefly, the integration of a hygroscopic hydrogel cooling layer can suppress the initial performance decline by 50.00–60.00%, 43.84–63.26%, 35.34–103.38%, and 43.75–63.57% for V_oc_, P_max_, FF, and efficiency, respectively.

(2)

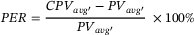

where *PV*_*avg*′_ and *CPV*_*avg*′_ are the average performance indicator data excluding the initial reading at 10 min of a pristine bifacial PV panel without cooling and a bifacial PV panel with a cooling layer, respectively (Table S3). The calculated PER results demonstrated that the hygroscopic hydrogel cooling layer can improve the steady‐state outputs for up to 4.86%, 12.40%, 3.44%, and 12.46% for V_oc_, P_max_, FF, and efficiency, respectively (Table S4). The instant‐cooling power was calculated based on Equation (3) using the hourly evaporated water measured by an electronic balance.

(3) $$P=\frac{\Delta H_{vap}\times \Delta m}{t\times A}$$

where Δ*H_vap_* is the enthalpy of vaporization of water (2,450 J g^−1^), Δ*m* is the weight loss due to water evaporation, t is the time interval for calculation (1800 s), and A is the area of the bifacial PV panel facing towards sunlight (26.24 cm^2^). Thus, the average cooling power provided by the PAM‐C3‐CaCl_2_@S hydrogel attached to both sides of the PV panel under 0.8, 1.0, and 1.2 kW m^−2^ solar irradiance was 441.8, 534.3, and 655.3 W m^−2^, respectively. The high cooling power originates from three synergistic factors: (1) bifacial hydrogel attachment doubles the evaporative cooling area; (2) the high water‐sorption capacity of 2.08 g g^−1^ at 90% RH ensures sufficient water for sustained evaporation; and (3) the SiO_2_ coating provides additional radiative cooling through phonon‐polariton resonances. These factors together account for the higher cooling power compared with conventional single‐side evaporative cooling systems.

### 2.4 Outdoor Field Tests

To evaluate the performance stability and capability under complicated real‐world environmental conditions, outdoor field tests were conducted on October 13 and October 15 of 2025 at the Meishan campus of Ningbo University (121° 57' 20“ E, 29° 46' 25” N), using PV panels with dimensions of 18 × 18 cm^2^. Testing was discontinued on October 14 to prevent potential damage to the sensors and devices during heavy rainfall. A variety of weather conditions were encountered, including clear skies, cloudy days, and rainfall. During the cloudy and rainy periods, the irradiance declined to near 0 W m^−2^ (Figure S51). Figure 4a,b depicted the outdoor field test site and the configuration of the experimental system, which contains essential sensors to measure the environmental conditions such as solar irradiance, wind speed, ambient temperature, and humidity. The charging case, coulomb meter, electronic balance, and thermocouple modules are employed to monitor the performance indicators of the PV panel and the hygroscopic hydrogel cooling layer; a communication hub was used to organize and transfer the recorded data to a data logging computer. The outdoor field test data of October 13, 2025, is presented in Figure 4c–f, while data of October 15, 2025, can be found in Note S5.

**FIGURE 4:**
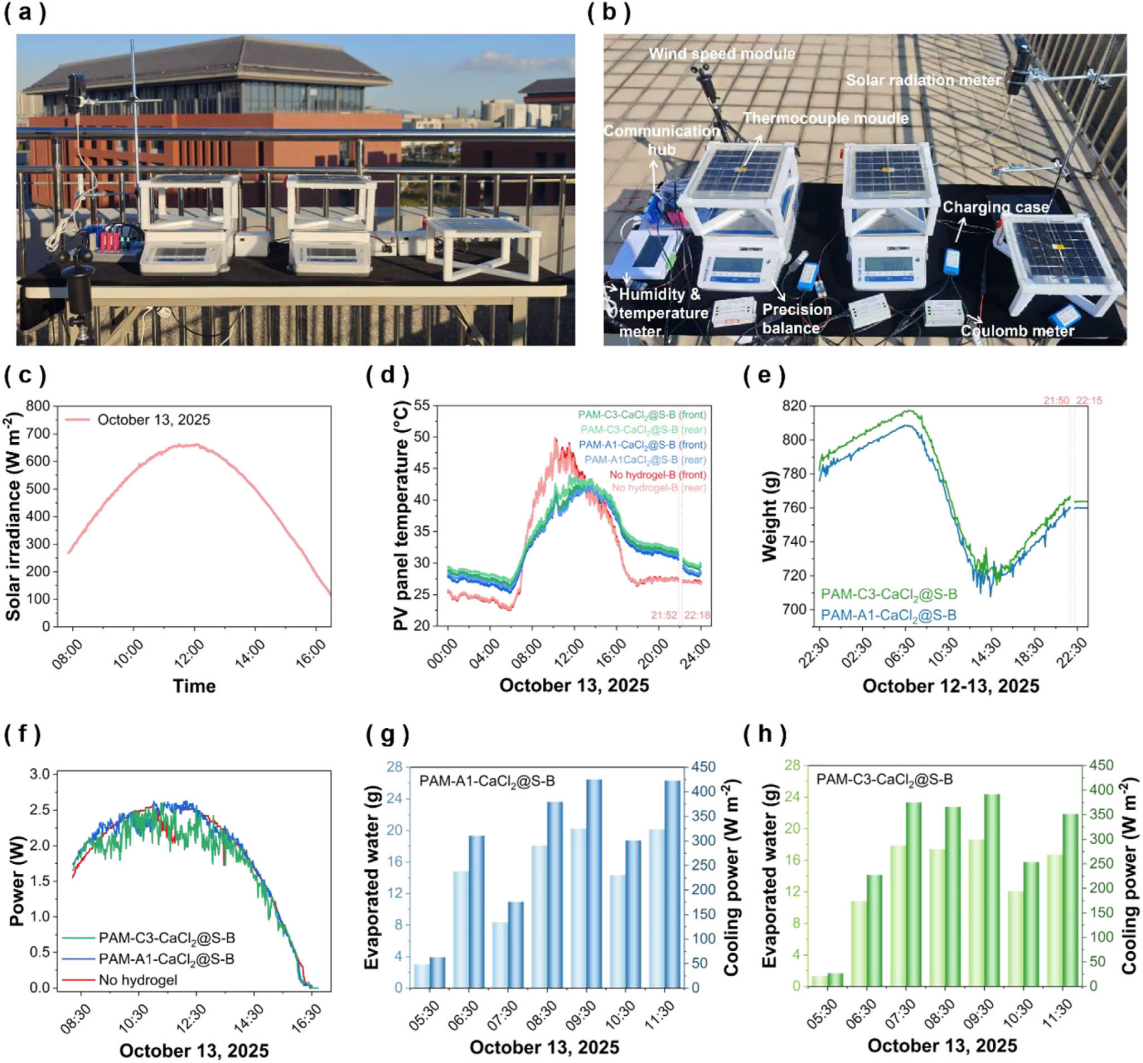
Outdoor field tests conducted at Ningbo University, Meishan Campus. (a) Digital photo of the experimental site. (b) Experimental configurations for outdoor field test. (c) Solar irradiance data of October 13, 2025. (d) Temperature profiles of the pristine bifacial PV panels, with the PAM‐C3‐CaCl_2_@S and PAM‐A1‐CaCl_2_@S cooling layers. (e) Weight change of the hygroscopic hydrogel cooling layer throughout the test on October 13. (f) P_max_ of bifacial PV panels during the test. (g) and (h) Cooling power and hourly weight loss of the hydrogel during the test.

The maximum solar irradiance on October 13, 2025 was approximately 660 W m^−2^ as the experiments were carried out in the late‐autumn season (Figure 4c). The highest temperature of bifacial PV panels was recorded at ∼10:10 am, which was ∼50°C for pristine panel without cooling and ∼41°C cooled by PAM‐C3‐CaCl_2_@S‐B and ∼40°C cooled by PAM‐A1‐CaCl_2_@S‐B. It should be noted that during nighttime, the temperature of the pristine PV panel was approximately 8°C lower than that of the panels with the attached hygroscopic hydrogel. This difference can be attributed to the release of latent heat during water vapor condensation and the high heat capacity of the water retained in the hydrogel (Figure 4d). The weight change profile of the hygroscopic hydrogels indicated that about 100 g of water evaporated during the daytime. After 3:00 p.m., the desorption process stopped, and water vapor sorption commenced, owing to insufficient solar irradiance and the consequent decrease in panel temperature resulting from the lack of solar‐to‐heat conversion (Figure 4e).

The instantaneous P_max_ enhancement of PAM‐C3‐CaCl_2_@S‐B and PAM‐A1‐CaCl_2_@S‐B reached up to 24.4% and 25.0%, respectively. The average enhancement, however, was calculated to be less than 5%, owing to the weak solar irradiance and the strong wind cooling effect typical of late autumn, which also indicates that better performance may be achievable in summer (Figure 4f and Figure S47). The peak cooling powers provided by the PAM‐A1‐CaCl_2_@S‐B and PAM‐C3‐CaCl_2_@S‐B during the test were 420 and 400 W m^−2^, respectively, determined from Equation (3) using the recorded weight of evaporated water. Both maxima were observed at 9:30 a.m., a time when the water content in the hydrogel remained ample, and evaporation proceeded readily due to the still‐low concentration of the hygroscopic salt solution retained within the hydrogel. The omniphobicity of the hydrogel remained unchanged after the outdoor field test compared with the freshly prepared sample. Contact angles greater than 150° for both water and mineral oil were observed for the PAM‐A1‐CaCl_2_@S and PAM‐C3‐CaCl_2_@S hydrogels following the outdoor field tests (Figure S61). Control evaluations of the three pristine PV panels were also conducted under identical outdoor field conditions to quantify their potential intrinsic differences (Figures S49 and S50). The results confirmed that the three panels employed in the field test exhibited negligible intrinsic differences. The unit price of the PAM‐A1‐CaCl_2_@S and PAM‐C3‐CaCl_2_@S were estimated to be $8.7 and $12.3 per m^2^, respectively, making them highly viable for practical applications (Table S5).

A COMSOL model was developed to investigate the cooling performance and water vapor evaporation during operation of an upscaled device within a three‐device array (Note S6). The simulation results showed that the influence of device upscaling on the cooling effect is negligible, although an edge effect was observed, where the edge of the PV panel exhibited a slightly higher temperature than the central regions. Humidity distributions revealed downstream drying due to wind‐carried heat; however, this effect had limited influence on the overall cooling performance across the entire device array. To further evaluate the applicability of this bifacial PV panel cooling strategy on the global scale, a predictive model was established based on the available geographical data and the PV market reports (Note S7). The prediction considered the annually available solar irradiance and average ambient humidity as key parameters, revealing that regions with abundant solar energy resources and sufficient ambient humidity, such as Mid‐Africa, South America, and South Asia, show the highest applicability. However, due to the substantially larger installed PV capacity in China and the United States, even though their ambient humidity and average solar irradiation are slightly lower than in low‐latitude countries, these countries demonstrate the highest carbon reduction potential at the country scale. The estimated carbon reduction potential contributions of China, the United States, Australia, and Brazil are significantly higher than those of other countries, with China and the United States alone representing 54% of the global total (Figure S68). In China and the U.S., CO_2_ reduction potentials reach 112.1 and 24.27 Mt in spring/winter, and 124.39 and 29.46 Mt in summer/autumn, respectively. The global carbon reduction potential was calculated to be 537.56 Mt annually, confirming that this strategy effectively boosts bifacial PV performance while making a meaningful contribution to global carbon reduction targets, especially in regions with large installed PV capacity such as China and the United States, which together represent over half of the estimated mitigation.

### 2.5 Environmental Implications and Scalability

While this work demonstrates promising cooling performance and considerable carbon mitigation potential, we acknowledge that a comprehensive environmental assessment should consider the full life cycle of the material. During fabrication, although organic solvents and energy input are involved, the material dosage per unit area is low and can be further reduced through scaled‐up manufacturing. During installation, the hydrogel is directly adhered to the PV panels without additional infrastructure. At end‐of‐life, the hydrogel can be peeled off and disposed of as chemical solid waste through established industrial channels. The main environmental concern lies in the fluorinated SiO_2_ coating, as perfluoroalkyl silanes fall within the broad PFAS definition. In this work, however, PFOES is covalently grafted onto SiO_2_ rather than physically adsorbed, and the dosage is very low; the coating remained intact after outdoor field tests, indicating negligible leaching during normal operation. Nevertheless, we fully recognize that the development of non‐fluorinated alternatives remains an important direction for future work.

In addition, the spin coating method used in this work, although ensuring precise control of coating thickness, is not suitable for industrial‐scale production. For future scale‐up, spray coating is a promising alternative, as it is compatible with large‐area PV panel manufacturing. The uniformity and functional performance of the coating can be ensured by optimizing key spray parameters, such as nozzle‐to‐substrate distance, flow rate, and substrate temperature. Automated spray systems with programmable motion can further improve reproducibility and consistency across large‐area substrates. These approaches merit further exploration.

## 3 Conclusions

In conclusion, this study successfully retards the photo‐oxidation process by quenching photo‐induced free radicals to fabricate a transparent, omniphobic, and hygroscopic hydrogel with superior UV resistance for zero‐energy‐demand bifacial PV cooling. Incorporating fullerene C_60_ into the hydrogel network effectively captures and quenches radicals, suppressing UV‐driven oxidative degradation and increasing UV stability by over 385%. The hydrogel harvests 2.08 g g^−1^ water at 90% RH. When applied to PV panels, passive daytime evaporative cooling is achieved using nighttime moisture sorption, yielding an average cooling power of 550–650 W m^−2^, a 14.3 °C temperature drop, and 9.03% gains in both efficiency and power output. Global deployment could reduce carbon emissions by 537.56 million metric tons annually. This design overcomes conventional cooling's energy and water penalties, offering an effective, maintenance‐free solution for bifacial PV systems.

## 4 Methods

### 4.1 Chemical and Materials

Acrylamide (AM, 99%), Silicon dioxide nanospheres (99.5%) were purchased from Macklin. N, N’‐Methylenebis (acrylamide) (MBAA, 99%), Potassium persulfate (KPS, 99.5%), N,N,N'N’‐Tetramethylethylenediamine (TEMED, 96%), Calcium chloride dihydrate (99%), Triethoxy‐1H,1H,2H,2H‐tridecafluoro‐n‐octylsilane (PFOES, 97%), 1‐Hexene (99%) were purchased from Aladdin. 3‐Aminopropyltriethoxysilane (APTES, 98%) was purchased from TCI Chemicals, Ethylacetate (99.5%) was purchased from Meryer. The above chemicals were used without further purification. Deionized water was used throughout the experiments. The photovoltaic panels with customized dimensions were purchased from Shanghai Sui‐Ying Co. Ltd.

### 4.2 Laboratory Conditions

Both the room temperature and relative humidity of the laboratory where the experiments were carried out were controlled by a heating, ventilation, and air‐conditioning (HVAC) system. The room temperature was maintained at 22°C, and the relative humidity was maintained at 60%.

### 4.3 Preparation of TAPC and TCPC

The preparation of amino/carboxyl fullerenes (TAPC/TCPC) followed previously reported procedures [46, 47]. Briefly, the synthesis of TAPC used hexachlorofullerene (C_60_Cl_6_) as a key precursor, which was dissolved in toluene, followed by sequential addition of anhydrous potassium carbonate, 80% cumene hydroperoxide, and 4‐(tert‐butoxycarbonylamino) piperidine. The mixture was stirred at room temperature for 1 h and was washed successively with saturated aqueous ammonium chloride and sodium bicarbonate solutions, dried over anhydrous sodium sulfate, and concentrated. The crude product was purified by silica gel column chromatography (toluene‐ethyl acetate system) to obtain the Boc‐protected tetraamino[60]fullerene epoxide. Subsequently, deprotection was carried out in dichloromethane with trifluoroacetic acid at room temperature for 2 h. The trifluoroacetate salt was converted to the hydrochloride salt using an ion‐exchange resin, and finally water‐soluble TAPC hydrochloride powder was obtained by freeze‐drying.

TCPC was prepared using fullerene C_60_ as the precursor. C_60_ and tert‐butyl 4‐piperidinecarboxylate were dissolved together in chlorobenzene, followed by addition of cumene hydroperoxide. The mixture was stirred at room temperature for 72 h until C_60_ was completely consumed. The reaction mixture was quenched with dimethyl sulfide, washed successively with saturated aqueous ammonium chloride and sodium bicarbonate solutions, dried over anhydrous sodium sulfate, and concentrated. The crude product was purified by silica gel column chromatography (toluene–ethyl acetate system) to obtain the tert‐butyl ester‐protected tetracarboxy[60]fullerene epoxide. Deprotection was carried out in 50% trifluoroacetic acid/chloroform at room temperature for 2 h to remove the tert‐butyl groups. The resulting product was dissolved in 100 µM sodium hydroxide solution to form the sodium salt, which was then dialyzed against ultrapure water for 48 h to yield pure TCPC sodium salt.

### 4.4 Preparation of Hydrophobic SiO_2_ Nanospheres

Briefly, 0.4 g of unmodified SiO_2_ particles with a diameter of 15 nm were added to a 1 wt.% PFOES‐Hexene solution. After thorough mixing, the container was left open and placed in a 50°C water bath inside a fume hood until the liquid had completely evaporated. The resulting hydrophobic silica particles are referred to as SiO_2_ in this report.

### 4.5 Preparation of PAM‐CaCl_2_ Hydrogel

1.92 g of acrylamide was fully dissolved in 9.16 mL of nitrogen‐purged CaCl_2_ solution, where the concentration of CaCl_2_ in the entire solution system was 0.40 g mL^−1^. Then, 40 µL of MBAA (8.33 µg mL^−1^), 400 µL of KPS (2.00 mg mL^−1^), and 4 µL of TEMED (0.32 µg mL^−1^) were sequentially added as the crosslinker, initiator, and catalyst, respectively. After mixing evenly, the mixture was allowed to stand to form a hydrogel.

### 4.6 Preparation of Fullerene‐embedded PAM‐CaCl_2_ Hydrogel

The preparation of fullerene‐embedded PAM‐CaCl_2_ hydrogel followed a similar procedure to that for the preparation of PAM‐CaCl_2_ hydrogel, except for the addition of TAPC/TCPC in the CaCl_2_ solution. The concentration of CaCl_2_ in the entire solution mixture was maintained at 0.4 g mL^−1^, and the concentration of TAPC/TCPC in the entire solution system was adjusted stepwise to 0.01, 0.03, 0.05, and 0.1 mg mL^−1^, respectively. The resulting hydrogels were denoted as PAM‐A1‐CaCl_2_ / PAM‐C1‐CaCl_2_, PAM‐A3‐CaCl_2_ / PAM‐C3‐CaCl_2_, PAM‐A5‐CaCl_2_ / PAM‐C5‐CaCl_2_, and PAM‐A10‐CaCl_2_ / PAM‐C10‐CaCl_2_, respectively.

### 4.7 Hydrogel Surface Modification

The as‐prepared hydrogels were immersed in a 5 wt.% APTES‐ethyl acetate solution for 20 s and subsequently removed and placed in a fume hood for 15 min to allow the liquid to evaporate. A SiO_2_‐ethyl acetate solution with a concentration of 5 mg mL^−1^ was prepared in advance and ultrasonicated for 20 min, then, 400 µL of the SiO_2_‐ethyl acetate solution was spin‐coated onto the hydrogel. This step was repeated 5 times until the hydrophobic SiO_2_ particles were uniformly coated onto the hydrogel, and the sample was denoted as PAM‐A_x_/C_x_‐CaCl_2_@S, where “x” indicated the concentration of fullerene embedded.

### 4.8 Material Characterizations

Scanning electron microscopy images and the energy‐dispersive x‐ray spectroscopy (EDS) Data were acquired using a Thermo Fisher Quattro S scanning electron microscope (SEM), equipped with an EDAX ELECT PIUS spectrometer. The Fourier‐transform infrared (FTIR) spectra were recorded on a Bruker Alpha II instrument. X‐ray diffraction (XRD) patterns were obtained using a Bruker D8 Advance DAVINCI diffractometer. X‐ray photoelectron spectroscopy (XPS) was conducted on a Thermo Fisher Nexsa system. The gravimetric dynamic water vapor sorption measurements were carried out with a Hiden IGSA250 dynamic vapor sorption analyzer. The contact angle measurements were performed using an Afes FCA2000‐A4 contact angle analyzer, with a droplet volume of 4 µL and a dropping rate of 1 µL/s, and the contact angles were determined using the three‐point method. The UV–vis absorption spectra were recorded on a PEREE TU‐1901 double‐beam ultraviolet‐visible spectrophotometer. The strain–stress testing was conducted on an Instron 3365 universal testing machine. A STIK ICTHI‐150 chamber was used for constant temperature and humidity control. Electron spin resonance (ESR) spectra were recorded on a Bruker E500 spectrometer. The elemental concentrations were measured by inductively coupled plasma optical emission spectrometry (ICP‐OES, Thermo Fisher iCAP 7000) and ion chromatography (IC, Qingdao Shenghan CIC‐D100).

### 4.9 PV Panel Cooling Test in Laboratory Conditions

The hydrogels were directly adhered to the glass encapsulation of the bifacial PV panels (front, rear, or both sides) through their self‐adhesive properties. The assembled device was placed on a 3D‐printed polylactic acid frame with customized dimensions, exposed under a solar simulator operated at different light intensities. The PV performances, including open‐circuit voltage (V_oc_), short‐circuit current (I_sc_), filling factor (FF, %), conversion efficiency, and maximum power output (P_max_) were measured using a Keithley 2400 source meter. The mass change of the hydrogels was recorded in real time using an electronic balance (Mettler Toledo MA1002E/A) connected to a computer for data logging. The temperature changes of the PV panels were recorded using an online thermal imager (Fotric 618C). The area of the solar cells in the PV panels used in the indoor experiments was 15.66 cm^2^.

### 4.10 Outdoor Field Tests

The effective area of the photovoltaic panel used in the outdoor experiment was 64.8 cm^2^. The hydrogel measured 18 × 18 × 0.3 cm^3^. The prepared hydrogel was tightly attached to both the front and rear surfaces of the bifacial PV panel, which was then placed outdoors to absorb moisture at night and cool the panel through water evaporation during the day. During the testing period, real‐time wind speed was recorded using a multifunctional anemometer (AM‐4836C), and light intensity variations were measured using a solar power meter (Tes, 1333R). Changes in ambient temperature and humidity were monitored with an RS‐485 temperature and humidity sensor (Sulink, CX03). The temperature of the bifacial PV panel was recorded using a thermocouple set (Kaipusen, KPS‐ZT‐TT‐K‐30‐2000‐CZ). The mass change of the hydrogel was continuously recorded by an electronic balance (METTLER TOLEDO MA1002E/A) connected to a computer (Acer, SQA27‐1300). The photovoltaic performance of the panel was recorded using a coulomb meter (Guichen GC90).

## Author Contributions

**Xiaoqi Zhou**: conceptualization, methodology, data curation, investigation, formal analysis, visualization, writing – original draft. **Chengyu Yang**: methodology, investigation, formal analysis, writing – original draft. **Rongrong Ma**: validation, writing – review and editing, methodology. **Kun Zhang**: investigation, validation. **Liyin Liu**: investigation. **Bo Wu**: resources, writing – review and editing, funding acquisition. **Renyuan Li**: conceptualization, supervision, funding acquisition, project administration, resources, writing – review and editing.

## Conflicts of Interest

R.L., X.Z., and R.M. have a patent application related to the work presented in this paper.

## Supporting information




**Supporting File**: advs78353‐sup‐0001‐SuppMat.docx.

## Data Availability

The data that support the findings of this study are available from the corresponding author upon reasonable request.

## References

[1] IEA , Global Energy Review 2025 (IEA, Paris, 2025), https://www.iea.org/reports/global‐energy‐review‐2025.

[2] N. Kannan and D. Vakeesan , “Solar Energy for Future World:—A Review,” Renewable and Sustainable Energy Reviews 62 (2016): 1092–1105, 10.1016/j.rser.2016.05.022.

[3] Y. Fan , J. Meng , H. Ye , P. Wang , Y. Wang , and Y. Wang , “Sustainability and Ecological Efficiency of Low‐Carbon Power System: A Concentrating Solar Power Plant in China,” Journal of Environmental Management 290 (2021): 112659, 10.1016/j.jenvman.2021.112659. 33894485

[4] J. Lv , J. Xie , A. G. A. Mohamed , et al., “Solar Utilization Beyond Photosynthesis,” Nature Reviews Chemistry 7 (2023): 91–105, 10.1038/s41570-022-00448-9. 37117911

[5] MIT News , Shining Brightly (2011) https://news.mit.edu/2011/energy‐scale‐part3‐1026.

[6] R. Li , M. Wu , S. Aleid , C. Zhang , W. Wang , and P. Wang , “An Integrated Solar‐Driven System Produces Electricity With Fresh Water and Crops in Arid Regions,” Cell Reports Physical Science 3, no. 3 (2022): 100781, 10.1016/j.xcrp.2022.100781.

[7] X. J. Shang , J. F. He , H. L. Wang , et al., “Effect of Built‐in Electric Field in Photovoltaic InAs Quantum Dot Embedded,” GaAs Solar Cell Applied Physics A 103 (2011): 335–341, 10.1007/s00339-010-6152-8.

[8] Ember , Global Electricity Review 2025 (Ember, 2025), https://ember‐energy.org/latest‐insights/global‐electricity‐review‐2025/.

[9] IEA , Renewables 2024, (IEA, Paris, 2024), https://www.iea.org/reports/renewables‐2024.

[10] G. Masson , M. De l’Epine , I. Kaizuka , and J. Okazaki , Trends in PV Applications 2025 (IEA‐PVPS, 2025), 10.69766/NCNN2417.

[11] M. A. Mikofski , R. Darawali , M. Hamer , A. Neubert , and J. Newmiller , “Bifacial Performance Modeling in Large Arrays,” in 2019 IEEE 46th Photovoltaic Specialists Conference (PVSC) (IEEE, 2019), 10.1109/PVSC40753.2019.8980572.

[12] Z. Li , Y. Chen , R. Guo , et al., “RETRACTED: Doubling Power Conversion Efficiency of Si Solar Cells,” Advanced Materials 36, no. 48 (2024): 2405724, 10.1002/adma.202405724. 39188194

[13] A. Makki , S. Omer , and H. Sabir , “Advancements in Hybrid Photovoltaic Systems for Enhanced Solar Cells Performance,” Renewable and Sustainable Energy Reviews 41 (2015): 658–684, 10.1016/j.rser.2014.08.069.

[14] J. Haschke , J. P. Seif , Y. Riesen , et al., “The Impact of Silicon Solar Cell Architecture and Cell Interconnection on Energy Yield in Hot & Sunny Climates,” Energy & Environmental Science 10 (2017): 1196–1206, 10.1039/C7EE00286F.

[15] L. Xu , W. Liu , H. Liu , et al., “Heat Generation and Mitigation in Silicon Solar Cells and Modules,” Joule 5, no. 3 (2021): 631–645, 10.1016/j.joule.2021.01.012.

[16] M. J. Huang , P. C. Eames , B. Norton , and N. J. Hewitt , “Natural Convection in an Internally Finned Phase Change Material Heat Sink for the Thermal Management of Photovoltaics,” Solar Energy Materials and Solar Cells 95, no. 7 (2011): 1598–1603, 10.1016/j.solmat.2011.01.008.

[17] P. Cheng , Z. Tang , X. Chen , et al., “Advanced Phase Change Hydrogel Integrating Metal‐Organic Framework for Self‐Powered Thermal Management,” Nano Energy 105 (2023): 108009, 10.1016/j.nanoen.2022.108009.

[18] X. Yu , M. Liang , C. Dong , and L.‐Z. Zhang , “Hygroscopic Hydrogel‐Based Cooling System for Photovoltaic Panels: An Experimental and Numerical Study,” Applied Thermal Engineering 257 (2024): 124400, 10.1016/j.applthermaleng.2024.124400.

[19] X. Liu , S. Yue , J. Li , and L. Lu , “Study of a Dust Deposition Mechanism Dominated by Electrostatic Force on a Solar Photovoltaic Module,” Science of the Total Environment 754 (2021): 142241, 10.1016/j.scitotenv.2020.142241. 33254918

[20] X. Li , J. Wang , and G. Su , “Electrostatic Force of Dust Deposition Originating from Contact between Particles and Photovoltaic Glass,” Chinese Physics B 30 (2021): 104101, 10.1088/1674-1056/abeb12.

[21] A. M. Elbreki , M. A. Alghoul , A. N. Al‐Shamani , et al., “The Role of Climatic‐Design‐Operational Parameters on Combined PV/T Collector Performance: A Critical Review,” Renewable and Sustainable Energy Reviews 57 (2016): 602–647, 10.1016/j.rser.2015.11.077.

[22] R. Talebnejad , S. Kamfiroozi , H. Ebadi , et al., “A New Cooling Method for Photovoltaic Panels Using Brine From Reverse Osmosis Units to Increase Efficiency and Improve Productivity,” Energy Conversion and Management 251 (2022): 115031, 10.1016/j.enconman.2021.115031.

[23] J. Kettle , M. Aghaei , S. Ahmad , et al., “Review of Technology Specific Degradation in Crystalline Silicon, Cadmium Telluride, Copper Indium Gallium Selenide, Dye Sensitised, Organic and Perovskite Solar Cells in Photovoltaic Modules: Understanding How Reliability Improvements in Mature Technologies Can Enhance Emerging Technologies,” Progress in Photovoltaics: Research and Applications 30 (2022): 1365–1392, 10.1002/pip.3577.

[24] D. Bredemeier , D. Walter , S. Herlufsen , and J. Schmidt , “Lifetime Degradation and Regeneration in Multicrystalline Silicon Under Illumination at Elevated Temperature,” AIP Advances 6, no. 3 (2016): 035119, 10.1063/1.4944839.

[25] K. A. Moharram , M. S. Abd‐Elhady , H. A. Kandil , and H. El‐Sherif , “Enhancing the Performance of Photovoltaic Panels by Water Cooling,” Ain Shams Engineering Journal 4, no. 4 (2013): 869–877, 10.1016/j.asej.2013.03.005.

[26] T. Montheil , C. Echalier , J. Martinez , G. Subra , and A. Mehdi , “Inorganic Polymerization: An Attractive Route to Biocompatible Hybrid Hydrogels,” Journal of Materials Chemistry B 6, no. 21 (2018): 3434–3448, 10.1039/C8TB00456K. 32254441

[27] S. Nižetić , A. M. Papadopoulos , and E. Giama , “Comprehensive Analysis and General Economic‐Environmental Evaluation of Cooling Techniques for Photovoltaic Panels, Part I: Passive Cooling Techniques,” Energy Conversation and Management 149 (2017): 334–354, 10.1016/j.enconman.2017.07.022.

[28] H. Dirawi , Q. Wang , M. Hu , Y. Su , and S. Riffat , “A Hydronic Closed‐Loop Photovoltaic Cooling System Designed for Hot and Arid Regions: Performance Evaluation and Degradation Rate/Lifetime Analysis,” Applied Energy 373 (2024): 123999, 10.1016/j.apenergy.2024.123999.

[29] Z. Aslam , S. I. U. H. Gilani , T. I. Mohamad , M. Muhammad , K. T. Alao , and M. Shaban , “Multi‐Methods Cooling Strategies for Concentrated Solar Power (CSP) Plants: Technologies, Challenges, and Opportunities,” Multiscale and Multidisciplinary Modeling, Experiments and Design 9, no. 1 (2026): 14, 10.1007/s41939-025-01080-y.

[30] R. Li , Y. Shi , M. Wu , S. Hong , and P. Wang , “Photovoltaic Panel Cooling by Atmospheric Water Sorption–Evaporation Cycle,” Nature Sustainability 3, no. 8 (2020): 636–643, 10.1038/s41893-020-0535-4.

[31] M. J. Caulfield , G. G. Qiao , and D. H. Solomon , “Some Aspects of the Properties and Degradation of Polyacrylamides,” Chemical Reviews 102, no. 9 (2002): 3067–3084, 10.1021/cr010439p. 12222982

[32] B. Xiong , R. D. Loss , D. Shields , et al., “Polyacrylamide Degradation and Its Implications in Environmental Systems,” npj Clean Water 1 (2018): 17, 10.1038/s41545-018-0016-8.

[33] Y. Hashikawa , H. Yasui , K. Kurotobi , and Y. Murata , “Synthesis and Properties of Open‐Cage Fullerene C_60_ Derivatives: Impact of the Extended π‐Conjugation,” Materials Chemistry Frontiers 2, no. 2 (2018): 206–213, 10.1039/C7QM00449D.

[34] E. B. Zeinalov and G. Koßmehl , “Fullerene C_60_ as an Antioxidant for Polymers,” Polymer Degradation and Stability 71, no. 2 (2001): 197–202, 10.1016/S0141-3910(00)00109-9.

[35] S. Osuna , M. Swart , and M. Solà , “On the Mechanism of Action of Fullerene Derivatives in Superoxide Dismutation,” Chemistry—A European Journal 16, no. 10 (2010): 3207–3214, 10.1002/chem.200902728. 20119990

[36] Z. Markovic and V. Trajkovic , “Biomedical Potential of the Reactive Oxygen Species Generation and Quenching by Fullerenes (C_60_),” Biomaterials 29, no. 26 (2008): 3561–3573, 10.1016/j.biomaterials.2008.05.005. 18534675

[37] W. Hou , L. Shen , Y. Zhu , et al., “Fullerene Derivatives for Tumor Treatment: Mechanisms and Application,” International Journal of Nanomedicine 19 (2024): 9771–9797, 10.2147/IJN.S476601. PMC11430870 39345909

[38] Z. Ding , X. Li , H. Zhang , et al., “Robust Radiative Cooling via Surface Phonon Coupling‐Enhanced Emissivity From SiO_2_ Micropillar Arrays,” International Journal of Heat and Mass Transfer 220 (2024): 125004, 10.1016/j.ijheatmasstransfer.2023.125004.

[39] I. S. Elashmawi and A. A. Al‐Muntaser , “Influence of Co_3_O_4_ Nanoparticles on the Optical, and Electrical Properties of CMC/PAM Polymer: Combined FTIR/DFT Study,” Journal of Inorganic and Organometallic Polymers and Materials 31, no. 6 (2021): 2682–2690, 10.1007/s10904-021-01956-9.

[40] K. Cheng , L. Zou , B. Chang , et al., “Mechanically Robust and Conductive Poly(acrylamide) Nanocomposite Hydrogel by the Synergistic Effect of Vinyl Hybrid Silica Nanoparticle and Polypyrrole for Human Motion Sensing,” Advanced Composites and Hybrid Materials 5, no. 4 (2022): 2834–2846, 10.1007/s42114-022-00465-8.

[41] H. S. Samanta and S. K. Ray , “Effect of Pectin and Attapulgite Filler on Swelling, Network Parameters and Controlled Release of Diltiazem Hydrochloride From Polyacrylic Copolymer Gel,” International Journal of Biological Macromolecules 190 (2021): 978–988, 10.1016/j.ijbiomac.2021.09.066. 34536479

[42] Y. Li , J. Xie , C. Li , and S. Cai , “Preparation and Properties of UV‐Curable PAA‐PAM Copolymer Hydrogels,” China Plast 39, no. 7 (2025): 22–27, 10.19491/j.issn.1001-9278.2025.07.005.

[43] J. Chen , L. Qiao , Z. Zhou , et al., “Promoted Hydroformylation of Formaldehyde By Electronic Metal–Support Interactions in N‐Group Functionalized Silica Supported Rhodium Catalyst,” Catalysis Letters 151, no. 12 (2021): 3664–3674, 10.1007/s10562-021-03568-x.

[44] C. Zhao , S. Zhang , C. Li , et al., “Study on the Quenching Mechanism of Radicals by Fullerenes (C_60_ and C_70_),” Inorganic Chemistry 65, no. 10 (2026): 5839–5849, 10.1021/acs.inorgchem.6c00397. 41760583

[45] L. Xiao , R. Huang , N. Sulimai , et al., “Amine Functionalized Trimetallic Nitride Endohedral Fullerenes: A Class of Nanoparticle to Tackle Low Back/Leg Pain,” ACS Applied Bio Materials 5, no. 6 (2022): 2943–2955, 10.1021/acsabm.2c00269. PMC9719410 35575694

[46] Y. Lu , J. Li , X. Liu , et al., “A Simple Synthesis of Tetraamino‐[60]Fullerene Epoxides as Potential Antitumor Drug Candidates,” CCS Chemistry 6, no. 10 (2024): 2392–2399, 10.31635/ccschem.024.202303784.

[47] J. Huo , J. Li , Y. Liu , et al., “Amphiphilic Aminated Derivatives of [60]Fullerene as Potent Inhibitors of Tumor Growth and Metastasis,” Advanced Science 9, no. 29 (2022): 2201541, 10.1002/advs.202201541. PMC9561876 36031401

