# Supplementary material for: Quenching Photo‐Induced Free Radicals Endows Superomniphobic Atmospheric Water Harvesting Hydrogels With UV Resistance for Durable Bifacial Photovoltaic Cooling
Source: Adv Sci (Weinh). 2026 Oct 22:e78353. Online ahead of print. doi: 10.1002/advs.78353 (PMC13690979; doi:10.1002/advs.78353)
Supplement: Supplementary file 1 [file ADVS-9999-e78353-s001.docx]

Supplementary Information

**Quenching photo-induced free radicals endows superomniphobic atmospheric water harvesting hydrogels with UV resistance for durable bifacial photovoltaic cooling**

Xiaoqi Zhou^1^, Chengyu Yang^1^, Rongrong Ma^1^, Kun Zhang^1^, Liyin Liu^1^, Bo Wu*^2,4,5^, and Renyuan Li*^1,3^

1. School of Marine Sciences, Ningbo University, Ningbo, 315832, P. R. China.

2. Beijing National Laboratory for Molecular Sciences, Key Laboratory of Molecular Nanostructure and Nanotechnology, Institute of Chemistry, Chinese Academy of Sciences, Beijing, 100190, China

3. School of Environmental Science and Technology, Key laboratory of Industrial Ecology and Environmental Engineering (Ministry of Education), Dalian University of Technology, Dalian, 116024, P. R. China.

4. University of Chinese Academy of Sciences, Beijing 100049, China

5. Center for Carbon Neutral Chemistry, Institute of Chemistry, Chinese Academy of Sciences, Beijing, 100190, China

* Corresponding authors.

Email: zkywubo@iccas.ac.cn; Lirenyuan@nbu.edu.cn

**Supplementary Note 1. Preliminary screening of TAPC and TCPC concentration in the hygroscopic hydrogels.**

Since functionalized fullerenes TAPC and TCPC play a major role in enhancing the UV aging resistance of the hygroscopic hydrogel, their concentration embedded in the material needs to be evaluated. In this section, a batch experiment was designed to test the UV tolerance of the hydrogel under high-dosage UV exposure in order to determine the optimal fullerene concentration. The concentrations of TAPC and TCPC were set to 0.01, 0.03, 0.05, and 0.1 mg mL^-1^. Hydrogel samples were cast and cut into 15 mm × 45 mm × 2 mm strips, placed in a UV aging chamber, and irradiated with a 50 W, 365 nm UV beam for a specified duration. The samples were subsequently cut into 1 mm × 1 mm × 2 mm strips for strain-stress testing using a universal testing machine. It should be noted that the 2 mm dimension refers to the sample thickness used specifically for mechanical testing, whereas the 3, 4, and 5 mm dimensions mentioned elsewhere refer to the hydrogel thickness used in the PV cooling tests, which were optimized by balancing cooling performance against material cost and weight. The 2 mm thickness was selected for mechanical testing because the hydrogel exhibits high extensibility, and thicker samples (e.g., 3 mm) could not be reliably fractured within the load capacity of the testing machine.

As shown in Figure S1 and S2, at the initial state (0 h of UV exposure), the pure PAM hydrogel exhibited significantly higher elongation at break (9862.39%) and tensile strength (0.331 MPa) than all PAM‑fullerene composite hydrogels, indicating that the introduction of fullerenes partially disrupted the crosslinking structure of the PAM network, leading to reduced initial mechanical properties. Nevertheless, certain fullerene‑embedded samples demonstrated superior UV aging resistance compared to pure PAM. After 1 h of UV exposure, the tensile strength of pure PAM drastically decreased to 0.072 MPa (a reduction of 78.17%), and its elongation at break decreased by 8.96%. In contrast, the elongation at break of PAM‑A1 (TAPC, 0.01 mg mL^-1^) and PAM‑C3 (TCPC, 0.03 mg mL^-1^) increased to 10760.18% and 9705.30%, respectively, corresponding to increases of 13.41% and 18.47% relative to their initial values, while their tensile strengths remained at relatively high levels of 0.151 MPa and 0.120 MPa. After 2 h of UV exposure, the mechanical properties of pure PAM continued to deteriorate, indicating irreversible damage to the network structure. Meanwhile, the elongation at break of PAM‑A1 increased to 19100.17%, with a tensile strength of 0.105 MPa, and PAM‑C1 and PAM‑C3 exhibited elongations at break as high as 14933.68% and 13389.06%, respectively. After 3 h of UV exposure, the mechanical structures of pure PAM collapsed, with elongation at break plummeting to 3650.65% and tensile strength to only 0.028 MPa, and even liquidized upon a 4-hour exposure. In contrast, PAM‑A1 still maintained a high elongation at break of 14046.92% and a tensile strength of 0.185 MPa, and PAM‑C3 also exhibited 10841.60% elongation at break and 0.203 MPa tensile strength. These results indicate that TAPC and TCPC at specific concentrations can effectively protect the polymer network structure and enhance mechanical stability under UV exposure.

Based on the mechanical performance evolution over 0-3 h, PAM‑A1, PAM‑C3, PAM‑C1, and PAM‑C10 all exhibited better UV aging resistance than pure PAM. However, in practical applications, the transparency of the hydrogel greatly affects the photovoltaic power generation efficiency, and the fullerene doping concentration is inversely correlated with transparency (Figure S3). The pure PAM‑CaCl_2_ hydrogel has a transparency of nearly 100%, whereas PAM‑A10/C10 (doped at 0.1 mg mL^-1^) has a cumulative transmittance of only ~85%, which is insufficient for photovoltaic panel requirements. In addition, although PAM‑C1 performed well at 2 h and 3 h, its mechanical properties at 0 h and 1 h were significantly weaker than those of PAM‑A1 and PAM‑C3. Therefore, considering both UV aging resistance and transparency, the optimal doping concentrations are 0.01 mg mL^-1^ for aminated fullerene (TAPC, corresponding to PAM‑A1) and 0.03 mg mL^-1^ for carboxylated fullerene (TCPC, corresponding to PAM‑C3), achieving the optimal anti‑aging performance under UV exposure.

**Figure S1.** Strain-stress curves of PAM and TAPC-embedded hydrogel at different UV exposure dosage.

**Figure S2.** Strain-stress curves of PAM and TCPC-embedded hydrogel at different UV exposure dosage.

**Figure S3.** UV-vis transmittance spectrum of PAM-CaCl_2_, PAM-A10-CaCl_2_, and PAM-C10-CaCl_2_.

**Figure S4.** SEM images of PAM-A1 and PAM-A1@S.

**Figure S5.** EDS survey mapping of element distributions of PAM-A1@S.

**Figure S6.** EDS mapping element distribution of F and Si in PAM-A1@S.

**Figure S7.** SEM images of PAM-C3 and PAM-C3@S.

**Figure S8.** EDS survey mapping of element distributions of PAM-C3@S.

**Figure S9.** EDS mapping element distribution of F and Si in PAM-C3@S.

**Figure S10.** High-resolution XPS spectra of PAM-A1@S and PAM-C3@S.

High-resolution spectra of F1s and Si2p further support the successful coating of the superomniphobic modification layer on the surfaces of samples PAM-A1 and PAM-C3. As shown in Figure S10a and S10c, the characteristic peaks at 688.51 eV (PAM-A1@S) and 688.39 eV (PAM-C3@S) are assigned to CF_2_, indicating the presence of fluorinated alkyl chains.^[1]^ An additional peak at 690.49 eV observed in PAM-C3@S corresponds to C-F units, suggesting the existence of highly fluorinated groups.^[2]^ The Si2p spectra exhibit peaks at 103.65 eV and 103.55 eV (Figure S10b and S10d), which are attributed to Si-O, indicating the silica nature of the hydrogel surface.^[3]^ Furthermore, the peaks at 105.74 eV and 107.79 eV are assigned to Si-F bonds,^[4]^ possibly originating from local fluorination of silicon sites during the modification process.

These observations confirm that the fluorinated species are chemically anchored onto the silicon surface. Additionally, the fluorinated silica nanoparticles are also physically adsorbed onto the hydrogel surface. These findings are consistent with the results obtained from contact angle and SEM characterization.

**Figure S11.** XRD patterns of SiO_2_ nano spheres.

**Figure S12.** Digital photo of PEOS coated glass slide.

**Figure S13.** Digital photo of PAM-A1‑CaCl_2_ and PAM-A1‑CaCl_2_@S hydrogel.

**Figure S14.** Digital photo of PAM-C3‑CaCl_2_ and PAM-C3‑CaCl_2_@S hydrogel.

**Figure S15.** Water and mineral oil contact angles of freshly prepared PAM-A1-CaCl_2_ hydrogel and PAM-A1-CaCl_2_@S hydrogel.

**Figure S16.** Salt retention of the PAM-CaCl_2_ hydrogel after 25 sorption-desorption cycles. Cl^-^ concentration measured by ion chromatography (IC) was 109.13 mg L^-1^ before cycling and 106.28 mg L^-1^ after 25 cycles, with a maximum relative change of approximately 3.5%, confirming that the salt remains confined within the polymer network.

**Figure S17.** Extended cycling stability of the same specimens after 11 months of storage.

**Supplementary Note 2. Mechanistic Study of Quenching Photo-induced Free Radicals by Functionalized Fullerenes.**

Prior to the calculations, washing control experiments were performed to verify the radical source. As shown in Figure S18, ICP-OES confirmed that the sulfur concentration in the unwashed PAM-H₂O hydrogel was 2.88 mg L^-1^, whereas that in the washed hydrogel was only 0.02 mg L^-1^. In addition, IC confirmed that the SO_4_^2-^ concentration in the solution before washing was 1.97 mg L^-1^, while that after washing was below the detection limit (0.1 mg L^-1^). As shown in Figure S19, ESR measurements revealed that the unwashed PAM-H_2_O hydrogel exhibited a strong and distinct hydroxyl radical signal after 20 min of UV irradiation, whereas the washed PAM-H_2_O hydrogel exhibited a much weaker but still discernible signal (Figure S20). These results indicate that residual KPS is the major radical source, while the PAM-water system itself also contributes a weak radical signal.

The density functional theory (DFT) and molecular dynamics (MD) calculations were carried out with the MS code.^[5]^ The Perdew-Burke-Ernzerhof (PBE) functional within generalized gradient approximation (GGA)^[6]^ was used to process the exchange-correlation, while the projector augmented-wave pseudopotential (PAW)^[7]^ was applied with a kinetic energy cut-off of 500 eV, which was utilized to describe the expansion of the electronic eigenfunctions. The Brillouin-zone integration was sampled by a Γ-centered 1 × 1 × 1 Monkhorst-Pack k-point. All atomic positions were fully relaxed until energy and force reached a tolerance of 1 × 10^-6^ eV and 0.01 eV Å^-1^, respectively. The dispersion corrected DFT-D method was employed to consider the long-range interactions.^[8]^ The model was established using Materials Studio software. During the molecular dynamics simulation, The NVT ensemble is used to relax the system, with a time step set to 1 fs and a total duration of 10 ns.

From the perspective of interaction energy, both fullerenes exhibit clear and consistent trends in their interactions with different species. For TAPC, the interaction energies with PAM, H_2_O, OH^-^, and ·OH are approximately -1.75, -0.72, -0.49, and -2.01 eV, respectively; for TCPC, the corresponding values are -1.88, -0.76, -0.54, and -2.08 eV. It is evident that, regardless of the fullerene type, the absolute value of the interaction energy with ·OH is the largest, significantly exceeding those with H_2_O and OH^-^, and even stronger than the interaction with the PAM matrix. This indicates that the fullerene surface exhibits the strongest adsorption/binding affinity toward hydroxyl radicals. In other words, once ·OH is generated in the PAM hydrogel system under UV irradiation, it is thermodynamically more favorable for the radicals to approach and bind to the fullerene surface rather than remain in a free state. Therefore, from an energetic standpoint, fullerenes inherently possess a “preferential capture” capability toward ·OH, which serves as the first-principles basis for their radical scavenging behavior.

**Figure S18.** Concentration of sulfur element of the unwashed and washed PAM-H_2_O hydrogels measured by ICP-OES.

**Figure S19.** ESR spectrum of the unwashed PAM-H_2_O hydrogel after 20 min of UV irradiation.

**Figure S20.** ESR spectrum of the washed PAM-H_2_O hydrogel after 20 min of UV irradiation.

**Figure S21.** DFT calculated interaction energy of TAPC with PAM, water, hydroxide ions, and hydroxyl radicals.

**Figure S22.** DFT calculated interaction energy of TCPC with PAM, water, hydroxide ions, and hydroxyl radicals.

Further analysis based on charge transfer provides more direct evidence supporting this conclusion. Notably, the charge transfer between fullerenes and ·OH is significantly higher than that with H_2_O and OH^-^, indicating that the interaction with ·OH is not merely physical adsorption but involves pronounced electronic redistribution and orbital coupling. Given that hydroxyl radicals possess unpaired electrons and extremely high reactivity, such substantial charge transfer suggests that fullerenes can act as electron-buffering and delocalization platforms. Through their π-conjugated framework, they can delocalize and stabilize the radical electrons, effectively “confining” and “passivating” the highly reactive ·OH species. This reflects the electronic-structure origin of radical quenching, fullerenes do not simply adsorb radicals, but reduce their spin activity and reactivity via electron acceptance/donation and π-conjugation-induced delocalization. This conclusion is further supported by the differential charge density distributions. In particular, for the highly reactive ·OH species, the C=C conjugated network on the fullerene surface provides ideal sites for addition and electron delocalization, enabling the formation of a surface-bound state that suppresses further attack on the PAM backbone or other components.

Meanwhile, our calculations also demonstrate that fullerenes can be stably incorporated into the PAM hydrogel system. The interaction energies between the two fullerenes and PAM reach -1.75 eV and -1.88 eV, with charge transfer values of 0.85 e and 0.88 e, respectively, indicating strong interfacial binding and electronic interactions between fullerenes and polyacrylamide chains. This suggests that fullerenes are not merely inert fillers dispersed in the system, but can be firmly anchored within the PAM network, forming stable radical-responsive sites. Consequently, under UV irradiation, once ·OH radicals are generated from H_2_O/OH^-^ in the hydrogel, they can be readily captured by nearby fullerenes and subsequently stabilized *via* the conjugated framework, thereby suppressing further oxidative degradation of the polymer chains.

Prior to UV irradiation, the system exhibits a relatively homogeneous distribution of polymer chains, water molecules, and hydroxide ions within the hydrogel network. When ·OH is introduced into the system, the MD simulations track its migration and capture by fullerene, demonstrating that ·OH preferentially accumulates around the fullerene moieties rather than remaining near the PAM backbone. After introducing functionalized fullerenes into the system, a noticeable change in radical distribution behavior is observed. Instead of remaining randomly dispersed, the generated radicals preferentially migrate toward and accumulate around the fullerene moieties. The MD configurations clearly show that radicals are localized in the vicinity of the fullerene surface rather than being distributed near the PAM backbone. This indicates that fullerenes act as effective radical trapping centers, altering the diffusion pathways and ultimate fate of radicals within the hydrogel. In other words, radicals transition from a delocalized, mobile state to a localized, fullerene-associated state upon incorporation of fullerenes (Figure S22).

**Figure S23.** Hydroxyl radical generation under UV illumination and radical annihilation upon fullerene embedment in the PAM-based hygroscopic hydrogel.

The generation and quenching mechanisms of reactive oxygen species (ROS) in the system can be clearly elucidated based on the frontier molecular orbital (HOMO/LUMO) analysis. For H_2_O, OH^-^, and ·OH, the calculated LUMO energies are -1.877, -3.124, and -3.814 eV, respectively, while their HOMO energies are -10.052, -6.775, and -6.023 eV, respectively. Notably, the HOMO levels of OH^-^ (-6.775 eV) and ·OH (-6.023 eV) are significantly higher than that of H_2_O (-10.052 eV), indicating that OH^-^ and H_2_O can act as electron donors under UV excitation. Upon UV irradiation, photogenerated holes (h⁺) can preferentially extract electrons from OH^-^ or H_2_O, leading to the formation of hydroxyl radicals (·OH). Meanwhile, the relatively high HOMO and low LUMO of ·OH (-6.023 and -3.814 eV) suggest that it is a highly reactive intermediate with both strong electron-donating and electron-accepting capability. The HOMO energies of TAPC and TCPC (-9.124 eV and -10.106 eV) are much lower than the LUMO of ·OH (-3.814 eV), indicating that reverse electron transfer from fullerene to the radical is energetically unfavorable, thereby reinforcing the role of fullerenes as electron acceptors.

**Figure S24.** The frontier molecular orbital (HOMO/LUMO) analysis of H_2_O, OH-, ·OH, TAPC, and TCPC.

**Supplementary Note 3. Parameter determination for bifacial PV panel cooling.**

This section identifies two key parameters that influence the evaporative cooling of bifacial PV panels and their potential for enhancing electricity generation. The first parameter is the thickness of the hygroscopic hydrogel, which determines the maximum water vapor uptake capacity and the available water content for evaporative cooling. A batch experiment was conducted by using hygroscopic PAM-CaCl_2_ hydrogel as example and setting hydrogel thicknesses of 3 mm, 4 mm, and 5 mm, attaching the hydrogel to the bifacial PV panel, and exposing the assembly under strong simulated solar irradiation at 1.2 kW m^-2^ (i.e*.*, 1.2 Sun) for cooling performance evaluation. The second parameter is the configuration of the hydrogel-PV panel assembly, specifically, the attachment location of the hydrogel to the bifacial PV panel, such as the front side, the rear side, or both sides, which influences the light path, thermal conductivity, and the total device weigh. The third parameter involved a comprehensive indoor variable experiment to compare the performance of 3mm-thick hydrogels, including PAM-CaCl_2_, PAM-CaCl_2_@S, PAM-A1-CaCl_2_, PAM-A1-CaCl_2_@S, PAM-C3-CaCl_2_, PAM-C3-CaCl_2_@S, Attached to the different side of bifacial PV panel under different light intensities.

The temperature profile of the pristine bifacial PV panel under 1.2 kW m^-2^ without cooling was determined as the reference temperature for comparison (Figure S25). As shown in Figure S26, when the hydrogels are attached to the both sides of the PV panel, the PAM-CaCl_2_ hydrogel with a thickness of 5 mm exhibited the best cooling performance, reducing the center temperature to 38.11 °C, which is 2.01 °C lower than that of the 3 mm PAM-CaCl_2_. This implies that greater thickness leads to better cooling performance due to superior available water content. However, as the number of testing cycles increased, the temperature difference between varied thicknesses gradually diminished. By the third cycle, the center temperature of the PV panel with 3mm PAM-CaCl_2_ hydrogel reached 37.85 °C, which was 0.46 °C and 0.78 °C lower than those of the 5 mm and 4 mm samples, respectively. This observation suggests that under continuous repeated sorption-desorption cycles, the 3 mm hydrogel provides sufficient cooling performance due to its excellent water vapor uptake capacity and favorable mass and heat transfer capabilities. Considering the weight limitations of the PV module and the reduced light transmittance associated with increased hydrogel thickness, 3 mm was determined to be the optimal thickness.

A further comparison of hydrogel attachment configurations revealed that the attachment of hygroscopic hydrogel cooling layer on both sides of the PV panels significantly outperformed front-side-only attachment. Taking the 3 mm hydrogel as an example, during the first cycle, the PV module with front-side-only attachment exhibited a center temperature of 42.93 °C, which was 2.8 °C higher than that with both-sides attachment. The same trend was observed in the second and third cycles, where the front-side-only attachment resulted in center temperatures 6.55 °C and 5.18 °C higher than those of the both-sides attachment configuration, respectively. Hydrogels of 4 mm and 5 mm thickness showed identical behavior, suggesting that the both-sides attachment configuration enables more complete utilization of the hydrogel’s evaporative cooling potential.
The results of this comprehensive indoor variable experiment revealed that the presence or absence of the silica film coating did not degrade the cooling performance of the hydrogel cooling system on the PV panels. On the contrary, the silica film may even provide superior radiative cooling performance due to the abundant Si-O-Si bonds on its surface. As shown in Figure S35, after three hours of illumination at light intensities of 0.8, 1.0, and 1.2  kW m^-2^, the center temperatures of the PV module with PAM-CaCl_2_-B were 35.0 °C, 38.3 °C, and 43.4 °C, respectively, which are 0-1.6 °C higher than those of PAM-CaCl_2_@S-B (Figure S38) (34.3 °C, 38.3 °C, and 41.8°C). Similar trends were observed for the other hydrogel groups. Furthermore, both sides attachment consistently outperformed front side and rear side configurations. As shown in Figure S33 and S34, the both sides attachment configuration achieved a center temperature up to 4.7 °C lower than front side attachment and up to 2.9 °C lower than rear side attachment, demonstrating that both sides attachment is the optimal assembly configuration.

**Figure S25.** Temperature profile of bifacial PV panel under 1.2 kW m^-2^ without cooling.

**Figure S26.** Temperature profiles of bifacial PV panel cooled with PAM-CaCl_2_ hydrogels with different thicknesses under 1.2 kW m^-2^. Cycle No. 1.

**Figure S27.** Temperature profiles of bifacial PV panel cooled with PAM-CaCl_2_ hydrogels with different thicknesses under 1.2 kW m^-2^. Cycle No. 2.

**Figure S28.** Temperature profiles of bifacial PV panel cooled with PAM-CaCl_2_ hydrogels with different thicknesses under 1.2 kW m^-2^. Cycle No. 3.

**Figure S29.** Temperature profiles of bifacial PV panel cooled by 3 mm PAM-A1-CaCl_2_@S, PAM-A1-CaCl_2_, PAM-C3-CaCl_2_@S and PAM-C3-CaCl_2_ hydrogel under 1.2 kW m^-2^ with different attachment configurations.

**Figure S30.** Bifacial PV panel cooling results in the laboratory conditions. (a) IR images of the bifacial PV panel under different solar irradiance. (b) Temperature profiles of the bifacial PV panels with and without the PAM-A1-CaCl_2_@S cooling layer. (c) V_oc_, (d) P_max_ (e) efficiency, and (f) FF of bifacial PV panel with or without cooling. (g) Real-time water evaporation profile during the test under different solar irradiance. (h) Cooling power (columns) and hourly-weight loss profiles (curves) of the hydrogel during the test.

**Figure S31.** Efficiency of bifacial PV panel with or without cooling by PAM-C3-CaCl_2_@S hydrogel with both-sides attachment configuration.

**Figure S32.** Fill factor of bifacial PV panel with or without cooling by PAM-C3-CaCl_2_@S hydrogel with both-sides attachment configuration.

**Table S1**. Performance degradation at first 10 min.

|  | | V_oc_ (V) | P_max_ (mW) | FF (%) | Efficiency (%) |
| --- | --- | --- | --- | --- | --- |
| 0.8 kW m^-2^ | PV | 0.24 | 9.99 | 2.07 | 0.80 |
|  | PAM-CaCl_2_@S-B | 0.1 | 5.61 | 0.78 | 0.45 |
|  | PAM-A1-CaCl_2_@S-B | 0.12 | 5.01 | 0.44 | 0.4 |
|  | PAM-C3-CaCl_2_@S-B | 0.12 | 4.52 | -0.07 | 0.36 |
| 1.0 kW m^-2^ | PV | 0.31 | 15.37 | 1.84 | 0.98 |
|  | PAM-CaCl_2_@S-B | 0.15 | 8.08 | 0.88 | 0.52 |
|  | PAM-A1-CaCl_2_@S-B | 0.14 | 6.58 | 0.61 | 0.42 |
|  | PAM-C3-CaCl_2_@S-B | 0.14 | 7.38 | 0.73 | 0.47 |
| 1.2 kW m^-2^ | PV | 0.40 | 24.28 | 2.49 | 1.29 |
|  | PAM-CaCl_2_@S-B | 0.16 | 8.92 | 0.63 | 0.47 |
|  | PAM-A1-CaCl_2_@S-B | 0.16 | 10.88 | 1.61 | 0.58 |
|  | PAM-C3-CaCl_2_@S-B | 0.20 | 11.85 | 1.37 | 0.63 |

**Table S2.** The calculated PDDR results.

|  | | V_oc_ (V) | P_max_ (mW) | FF (%) | Efficiency (%) |
| --- | --- | --- | --- | --- | --- |
| 0.8 kW m^-2^ | PAM-CaCl_2_@S-B | 58.33% | 43.84% | 62.32% | 43.75% |
|  | PAM-A1-CaCl_2_@S-B | 50.00% | 49.85% | 78.74% | 50.00% |
|  | PAM-C3-CaCl_2_@S-B | 50.00% | 54.75% | 103.38% | 55.00% |
| 1.0 kW m^-2^ | PAM-CaCl_2_@S-B | 51.61% | 47.43% | 52.17% | 46.94% |
|  | PAM-A1-CaCl_2_@S-B | 54.84% | 57.19% | 66.85% | 57.14% |
|  | PAM-C3-CaCl_2_@S-B | 54.84% | 51.98% | 60.33% | 52.04% |
| 1.2 kW m^-2^ | PAM-CaCl_2_@S-B | 60.00% | 63.26% | 74.70% | 63.57% |
|  | PAM-A1-CaCl_2_@S-B | 60.00% | 55.19% | 35.34% | 55.04% |
|  | PAM-C3-CaCl_2_@S-B | 50.00% | 51.19% | 44.98% | 51.16% |

**Table S3.** The average performance indicator data excluding the initial reading at 10 min.

|  | | V_oc_ (V) | P_max_ (mW) | FF (%) | Efficiency (%) |
| --- | --- | --- | --- | --- | --- |
| 0.8 kW m^-2^ | PV | 5.79 | 153.87 | 81.12 | 12.28 |
|  | PAM-CaCl_2_@S-B | 5.98 | 170.43 | 81.29 | 13.6 |
|  | PAM-A1-CaCl_2_@S-B | 5.93 | 166.13 | 81.71 | 13.26 |
|  | PAM-C3-CaCl_2_@S-B | 5.96 | 163.99 | 81.95 | 13.09 |
| 1.0 kW m^-2^ | PV | 5.79 | 201.15 | 80.15 | 12.84 |
|  | PAM-CaCl_2_@S-B | 6.01 | 226.10 | 80.90 | 14.44 |
|  | PAM-A1-CaCl_2_@S-B | 5.99 | 219.31 | 81.12 | 14.00 |
|  | PAM-C3-CaCl_2_@S-B | 6.01 | 217.07 | 81.33 | 13.86 |
| 1.2 kW m^-2^ | PV | 5.76 | 249.38 | 79.35 | 13.27 |
|  | PAM-CaCl_2_@S-B | 6.04 | 270.87 | 80.50 | 14.41 |
|  | PAM-A1-CaCl_2_@S-B | 6.02 | 266.00 | 80.92 | 14.16 |
|  | PAM-C3-CaCl_2_@S-B | 6.03 | 262.92 | 82.08 | 13.99 |

**Table S4.** The calculated PER results.

|  | | V_oc_ (V) | P_max_ (mW) | FF (%) | Efficiency (%) |
| --- | --- | --- | --- | --- | --- |
| 0.8 kW m^-2^ | PAM-CaCl_2_@S-B | 3.28% | 10.76% | 0.21% | 10.75% |
|  | PAM-A1-CaCl_2_@S-B | 2.42% | 7.97% | 0.73% | 7.98% |
|  | PAM-C3-CaCl_2_@S-B | 2.94% | 6.58% | 1.02% | 6.60% |
| 1.0 kW m^-2^ | PAM-CaCl_2_@S-B | 3.80% | 12.40% | 0.94% | 12.46% |
|  | PAM-A1-CaCl_2_@S-B | 3.45% | 9.03% | 1.21% | 9.03% |
|  | PAM-C3-CaCl_2_@S-B | 3.80% | 7.91% | 1.47% | 7.94% |
| 1.2 kW m^-2^ | PAM-CaCl_2_@S-B | 4.86% | 8.62% | 1.45% | 8.59% |
|  | PAM-A1-CaCl_2_@S-B | 4.51% | 6.66% | 1.98% | 6.71% |
|  | PAM-C3-CaCl_2_@S-B | 4.69% | 5.43% | 3.44% | 5.43% |

**Supplementary Note 4. Bifacial PV panel cooling performance of different samples and hydrogel-PV panel configurations.**

Note: the remarks after the short dash of the hydrogel sample name denoted the hydrogel-PV panel configurations. F: hydrogel attached to the front side of bifacial PV panel, R: hydrogel attached to the rear side of bifacial PV panel, and B: hydrogel attached to both sides of bifacial PV panel.

**Figure S33.** Bifacial PV panel cooling results in the laboratory conditions of **PAM-CaCl_2_-F**. (a) IR images of the bifacial PV panel under different solar irradiance. (b) Temperature profiles of the bifacial PV panels with and without cooling layer. (c) V_oc_, (d) P_max_ (e) efficiency, and (f) FF of bifacial PV panel with or without cooling. (g) Real-time water evaporation profile during the test under different solar irradiance. (h) Cooling power (columns) and hourly-weight loss profiles (curves) of the hydrogel during the test.

**Figure S34.** Bifacial PV panel cooling results in the laboratory conditions of **PAM-CaCl_2_-R**. (a) IR images of the bifacial PV panel under different solar irradiance. (b) Temperature profiles of the bifacial PV panels with and without cooling layer. (c) V_oc_, (d) P_max_ (e) efficiency, and (f) FF of bifacial PV panel with or without cooling. (g) Real-time water evaporation profile during the test under different solar irradiance. (h) Cooling power (columns) and hourly-weight loss profiles (curves) of the hydrogel during the test.

**Figure S35.** Bifacial PV panel cooling results in the laboratory conditions of **PAM-CaCl_2_-B.** (a) IR images of the bifacial PV panel under different solar irradiance. (b) Temperature profiles of the bifacial PV panels with and without cooling layer. (c) V_oc_, (d) P_max_ (e) efficiency, and (f) FF of bifacial PV panel with or without cooling. (g) Real-time water evaporation profile during the test under different solar irradiance. (h) Cooling power (columns) and hourly-weight loss profiles (curves) of the hydrogel during the test.

**Figure S36.** Bifacial PV panel cooling results in the laboratory conditions of **PAM-CaCl_2_@S-F**. (a) IR images of the bifacial PV panel under different solar irradiance. (b) Temperature profiles of the bifacial PV panels with and without cooling layer. (c) V_oc_, (d) P_max_ (e) efficiency, and (f) FF of bifacial PV panel with or without cooling. (g) Real-time water evaporation profile during the test under different solar irradiance. (h) Cooling power (columns) and hourly-weight loss profiles (curves) of the hydrogel during the test.

**Figure S37.** Bifacial PV panel cooling results in the laboratory conditions of **PAM-CaCl_2_@S-R**. (a) IR images of the bifacial PV panel under different solar irradiance. (b) Temperature profiles of the bifacial PV panels with and without cooling layer. (c) V_oc_, (d) P_max_ (e) efficiency, and (f) FF of bifacial PV panel with or without cooling. (g) Real-time water evaporation profile during the test under different solar irradiance. (h) Cooling power (columns) and hourly-weight loss profiles (curves) of the hydrogel during the test.

**Figure S38.** Bifacial PV panel cooling results in the laboratory conditions of **PAM-CaCl_2_@S-B**. (a) IR images of the bifacial PV panel under different solar irradiance. (b) Temperature profiles of the bifacial PV panels with and without cooling layer. (c) V_oc_, (d) P_max_ (e) efficiency, and (f) FF of bifacial PV panel with or without cooling. (g) Real-time water evaporation profile during the test under different solar irradiance. (h) Cooling power (columns) and hourly-weight loss profiles (curves) of the hydrogel during the test.

**Figure S39.** Bifacial PV panel cooling results in the laboratory conditions of **PAM-C3-CaCl_2_-F**. (a) IR images of the bifacial PV panel under different solar irradiance. (b) Temperature profiles of the bifacial PV panels with and without cooling layer. (c) V_oc_, (d) P_max_ (e) efficiency, and (f) FF of bifacial PV panel with or without cooling. (g) Real-time water evaporation profile during the test under different solar irradiance. (h) Cooling power (columns) and hourly-weight loss profiles (curves) of the hydrogel during the test.

**Figure S40.** Bifacial PV panel cooling results in the laboratory conditions of **PAM-C3-CaCl_2_-R**. (a) IR images of the bifacial PV panel under different solar irradiance. (b) Temperature profiles of the bifacial PV panels with and without cooling layer. (c) V_oc_, (d) P_max_ (e) efficiency, and (f) FF of bifacial PV panel with or without cooling. (g) Real-time water evaporation profile during the test under different solar irradiance. (h) Cooling power (columns) and hourly-weight loss profiles (curves) of the hydrogel during the test.

**Figure S41.** Bifacial PV panel cooling results in the laboratory conditions of **PAM-C3-CaCl_2_-B**. (a) IR images of the bifacial PV panel under different solar irradiance. (b) Temperature profiles of the bifacial PV panels with and without cooling layer. (c) V_oc_, (d) P_max_ (e) efficiency, and (f) FF of bifacial PV panel with or without cooling. (g) Real-time water evaporation profile during the test under different solar irradiance. (h) Cooling power (columns) and hourly-weight loss profiles (curves) of the hydrogel during the test.

**Figure S42.** Bifacial PV panel cooling results in the laboratory conditions of **PAM-C3-CaCl_2_@S-F**. (a) IR images of the bifacial PV panel under different solar irradiance. (b) Temperature profiles of the bifacial PV panels with and without cooling layer. (c) V_oc_, (d) P_max_ (e) efficiency, and (f) FF of bifacial PV panel with or without cooling. (g) Real-time water evaporation profile during the test under different solar irradiance. (h) Cooling power (columns) and hourly-weight loss profiles (curves) of the hydrogel during the test.

**Figure S43.** Bifacial PV panel cooling results in the laboratory conditions of **PAM-C3-CaCl_2_@S-R**. (a) IR images of the bifacial PV panel under different solar irradiance. (b) Temperature profiles of the bifacial PV panels with and without cooling layer. (c) V_oc_, (d) P_max_ (e) efficiency, and (f) FF of bifacial PV panel with or without cooling. (g) Real-time water evaporation profile during the test under different solar irradiance. (h) Cooling power (columns) and hourly-weight loss profiles (curves) of the hydrogel during the test.

**Figure S44.** Bifacial PV panel cooling results in the laboratory conditions of **PAM-A1-CaCl_2_-F**. (a) IR images of the bifacial PV panel under different solar irradiance. (b) Temperature profiles of the bifacial PV panels with and without cooling layer. (c) V_oc_, (d) P_max_ (e) efficiency, and (f) FF of bifacial PV panel with or without cooling. (g) Real-time water evaporation profile during the test under different solar irradiance. (h) Cooling power (columns) and hourly-weight loss profiles (curves) of the hydrogel during the test.

**Figure S45.** Bifacial PV panel cooling results in the laboratory conditions of **PAM-A1-CaCl_2_-R**. (a) IR images of the bifacial PV panel under different solar irradiance. (b) Temperature profiles of the bifacial PV panels with and without cooling layer. (c) V_oc_, (d) P_max_ (e) efficiency, and (f) FF of bifacial PV panel with or without cooling. (g) Real-time water evaporation profile during the test under different solar irradiance. (h) Cooling power (columns) and hourly-weight loss profiles (curves) of the hydrogel during the test.

**Figure S46.** Bifacial PV panel cooling results in the laboratory conditions of **PAM-A1-CaCl_2_-B**. (a) IR images of the bifacial PV panel under different solar irradiance. (b) Temperature profiles of the bifacial PV panels with and without cooling layer. (c) V_oc_, (d) P_max_ (e) efficiency, and (f) FF of bifacial PV panel with or without cooling. (g) Real-time water evaporation profile during the test under different solar irradiance. (h) Cooling power (columns) and hourly-weight loss profiles (curves) of the hydrogel during the test.

**Figure S47.** Bifacial PV panel cooling results in the laboratory conditions of **PAM-A1-CaCl_2_@S-F**. (a) IR images of the bifacial PV panel under different solar irradiance. (b) Temperature profiles of the bifacial PV panels with and without cooling layer. (c) V_oc_, (d) P_max_ (e) efficiency, and (f) FF of bifacial PV panel with or without cooling. (g) Real-time water evaporation profile during the test under different solar irradiance. (h) Cooling power (columns) and hourly-weight loss profiles (curves) of the hydrogel during the test.

**Figure S48.** Bifacial PV panel cooling results in the laboratory conditions of **PAM-A1-CaCl_2_@S-R**. (a) IR images of the bifacial PV panel under different solar irradiance. (b) Temperature profiles of the bifacial PV panels with and without cooling layer. (c) V_oc_, (d) P_max_ (e) efficiency, and (f) FF of bifacial PV panel with or without cooling. (g) Real-time water evaporation profile during the test under different solar irradiance. (h) Cooling power (columns) and hourly-weight loss profiles (curves) of the hydrogel during the test.

**Supplementary Note 5. Outdoor field test**

**Figure S49.** Outdoor conditions on November 14, 2025.

**Figure S50.** Performance of PV panels without hydrogel cooling on November 14, 2025.

**Figure S51.** Solar irradiation on October 15, 2025.

**Figure S52.** Ambient relative humidity of October 13 and 15, 2025.

**Figure S53.** Ambient temperature of October 13 and 15, 2025.

**Figure S54.**Wind speed data of October 13, 2025.

**Figure S55.**Wind speed data of October 15, 2025.

**Figure S56.** Temperature profiles of the pristine bifacial PV panels, with the PAM-C3-CaCl_2_@S and PAM-A1-CaCl_2_@S cooling layer.

**Figure S57.** Weight change of hygroscopic hydrogel cooling layer throughout the test on October 15.

**Figure S58.** Cooling power and hourly-weight loss of the PAM-A1-CaCl_2_@S hydrogel during the test on October 15, 2025.

**Figure S59.** Cooling power and hourly-weight loss of the PAM-C3-CaCl_2_@S hydrogel during the test on October 15, 2025.

**Figure S60.** P_max_ of bifacial PV panels during the test on October 15, 2025.

**Figure S61.** Water and mineral oil contact angles of PAM-A1-CaCl_2_@S and PAM-C3-CaCl_2_@S hydrogel after the outdoor field test.

**Table S5.** Prices of materials and chemicals for cost estimation.

| Material or Chemical | Source | CAS No. | Amount per m^2^ | Unit Price ($) | Cost per m^2^ |
| --- | --- | --- | --- | --- | --- |
| Acrylamide (AM, 99%) | Commercial | 79-06-1 | 1224.9600 g | 0.7337 kg^-1^ | 0.8988 |
| N,N′-Methylenebis (acrylamide) (MBAA, 99%) | Commercial | 110-26-9 | 0.0510 g | 5.1360 kg^-1^ | 0.0003 |
| Potassium persulfate (KPS, 99.5%) | Commercial | 7727-21-1 | 12.2496 g | 2.7881 kg^-1^ | 0.0341 |
| N,N,N’N’-Tetramethylethylenediamine (TEMED, 96%) | Commercial | 110-18-9 | 2.5520 mL | 0.2509 mL^-1^ | 0.6404 |
| Calcium chloride dihydrate (99%) | Commercial | 10035-04-8 | 2448.6440 g | 0.0514 kg^-1^ | 0.1258 |
| TAPC^*^ | Lab‑synthesized |  | 0.0612 g | 29348.3205 kg^-1^ | 1.7975 |
| TCPC^*^ | Lab‑synthesized |  | 0.1837 g | 29348.3205 kg^-1^ | 5.3926 |
| 15 nm SiO_2_ Nanoparticles Disperation^*^ | Lab‑synthesized |  | 2.0000 mL | 2.4521 mL^-1^ | 4.9047 |
| APTES-Ethylacetate Solution^*^ | Lab‑synthesized |  |  | 0.2729 m² | 0.2734 |

Price (PAM-A1-CaCl_2_@S) =∑Cost per m^2^ _(TAPC)_= $ 8.6570

Price (PAM-C3-CaCl_2_@S) =∑Cost per m^2^ _(TCPC)_= $ 12.2701

Note: Commercial prices were sourced from the Alibaba (https://www.1688.com/). Asterisks denote chemicals (^*^) were synthesized in Laboratory. The reported cost for these items includes raw materials, labor, energy, and facility overheads under laboratory scale. It should be noted that the production cost is expected to decrease substantially when scaled up to industrial manufacturing.

**Supplementary Note 6. COMSOL Multiphysics simulation**

A COMSOL model was developed to explore the intricate interactions among environmental conditions, solar irradiance, cooling performance, and related parameters, implemented at an upscaled device level and in a three‑device array configuration (Figure S62). In the operation of the AWH-DSC, the key factors governing water evaporation from the hydrogel layer are heat transfer and mass transfer. Owing to the small temperature differences among the PV panel, the hydrogel layer, and the ambient environment, the cooling effect of thermal conduction is neglected. The heat balance in the model is described as follows:

$$\begin{aligned} \text{Q}_{\text{in}}\text{=}\text{Q}_{\text{rad}\text{, }\text{PV}}\text{+}\text{Q}_{\text{conv}\text{, }\text{PV}}\text{+}\text{Q}_{\text{rad}\text{,h}\text{ydrogel}}\text{+}\text{Q}_{\text{conv}\text{, h}\text{ydrogel}}\text{+}{\text{∆}\text{H}}_{\text{Vap}}\text{∙}\text{f}_{\text{evap}}\#\text{(}\text{S}\text{1)} \end{aligned}$$

Where $\text{Q}_{\text{in}}$ is the heat power generated by bifacial PV panel during operation (W), $\text{Q}_{\text{rad}\text{, }\text{PV}}$, $\text{Q}_{\text{rad}\text{,h}\text{ydrogel}}$, $\text{Q}_{\text{conv}\text{, }\text{PV}}$, $\text{Q}_{\text{conv}\text{, h}\text{ydrogel}}$ are the heat exchange powers generated by radiation and thermal convection on the PV panel surface and the hydrogel surface, respectively (W). ${\text{∆}\text{H}}_{\text{Vap}}$ is the enthalpy of desorption of water from hydrogel, which is assumed to be equal to the latent heat of water (J g^-1^). $\text{f}_{\text{evap}}$ is the water vapor flux during evaporative cooling of bifacial PV panels (g s^-1^).

$$\begin{aligned} \text{Q}_{\text{rad}}\text{=}\text{Aεσ}\text{∙}\left( \text{T}_{\text{s}}^{\text{4}}\text{-}\text{T}_{\text{amb}}^{\text{4}} \right)\#\text{(}\text{S}\text{2)} \end{aligned}$$

Where $\text{Q}_{\text{rad}}$ is the radiative heat exchange power, A is the surface area of either PV panel or hydrogel (m^2^), $\text{ε}$ is the Stefan-Boltzmann constant (5.67ⅹ10^-8^ W·m^-2^·K^4^), $\text{σ}$ is the emissivity of hydrogel or PV panel, $\text{T}_{\text{S}}$ and $\text{T}_{\text{amb}}$ are the surface temperature of PV panel or hydrogel and the ambient (K).

$$\begin{aligned} \text{Q}_{\text{conv}}\text{=}\text{ρ}\text{C}_{\text{p}}\frac{\text{∂}\text{T}}{\text{∂}\text{t}}\text{+∇∙}\left( \text{-}\text{k}\text{∇}\text{T} \right)\text{+}\text{ρ}\text{C}_{\text{p}}\text{∙}\text{u}\text{∙∇}\text{T}\#\left( \text{S}\text{3} \right) \end{aligned}$$

Where $\text{Q}_{\text{conv}}$ is the convective heat exchange power, $\text{ρ}$ is the density of air (g m^-3^), $\text{C}_{\text{p}}$ is the heat capacity of air (J g^-1^ K^-1^), $\text{T}$ is temperature (K), $\text{t}$ is time (s), $\text{k}$ is the heat conductivity of air (W m^-2^ K), $\text{∇}\text{T}$ is the temperature gradient between surfaces of hydrogel or PV panel and the ambient air, $\text{u}$ is the velocity of air (m s^-1^). The transportation of water vapor can be expressed as:

$$\begin{aligned} \text{G}\text{=}\text{M}_{\text{v}}\frac{\text{∂}\text{C}_{\text{v}}}{\text{∂}\text{t}}\text{+}\text{M}_{\text{v}}\text{∙}\text{u}\text{∙∇}\text{C}_{\text{v}}\text{+∇∙(-}\text{D}\text{∇}\text{C}_{\text{v}}\text{)}\#\left( S4 \right) \end{aligned}$$

Where $\text{G}$ is the source term, $\text{M}_{\text{v}}$ is the molecular weight of water (g mol^-1^), $\text{C}_{\text{v}}$ is the concentration of water vapor (mol m^-3^), $\text{∇}\text{C}_{\text{v}}$ is the concentration gradient of water vapor. $\text{D}$ is the diffusion coefficient of water vapor in the ambient air (m^2^ s^-1^). Given that water constitutes the majority of a hydrogel’s composition, this study simplifies the calculation by treating the hydrogel as water, which has a saturated vapor pressure $\text{C}_{\text{sat}}$ at its surface temperature $\text{T}$. The following parameters were used for simulation:

(1) Dimension of the PV panel: 2 m ⅹ 1 m, 15^o^ titled towards wind filed, conversion efficiency 25%.

(2) Ambient temperature and humidity: 37 ℃, RH 50%

(3) Solar irradiance: 1.0 kW m^-2^

(4) Wind speed: 0.5 m s^-1^

**Figure S62.** Schematic of the dimension, device configuration, and wind field of the model. (a) Pristine bifacial PV panel without cooling, (b) front-side configuration, (c) rear-side configuration, and (d) both-sides configuration.

As seen, the pristine bifacial PV panel exhibited the highest equilibrium temperature of approximately 50 °C, whereas the configuration with hygroscopic hydrogels attached to both sides of the panel showed the lowest temperature, around 30 °C. The front‑side and rear‑side configurations yielded similar device temperatures, both higher than that of the both-side configuration but still substantially lower than that of the pristine PV panel under the same conditions. In all four configurations, the panel facing directly into the wind field exhibited the highest device temperature. Moreover, the edges of the panels showed lower temperatures than the central regions, owing to stronger convective cooling at the edges. The humidity distributions revealed the relative humidity fluctuations during operation. As shown in Figure S63a, the regions immediately downstream of the panels exhibited reduced relative humidity as the ambient temperature increased, due to the heat carried by the wind from the PV panels. The front‑side and rear‑side configurations displayed increased humidity resulting from the release of water vapor from the hydrogel, and also indicated the locations where water vapor was released (Figure S63b and S63c). For the both‑sides configuration, a greater amount of water can be released within the same time frame, which dissipates more energy and therefore results in the lowest device temperature (Figure S63d). An obvious wind‑driven suppression of water vapor field was also observed on the panel surfaces facing directly into the wind field, as the forced airflow rapidly removes the water vapor boundary layer above the hydrogel, thereby reducing the local partial pressure of water vapor and limiting further evaporation.

**Figure S63.** Simulation results of bifacial PV panel cooling effects at upscaled dimensions and different hydrogel-PV panel assembly configurations.

**Supplementary Note 7. Global carbon reduction potential (GCRP) prediction using Google Earth Engine (GEE)**

Before performing the simulation calculations, considering the differences in solar radiation across different seasons, the months from June to November were collectively defined as the summer and autumn season, while the remaining months were defined as the spring and winter season. The differences in solar radiation and anbient temperature between seasons are presented in Figure S64 and S65 (data source: ERA5-Land monthly averaged data from 1950 to present), and the radiation data for different regions were extracted for simulation calculations. Higher radiation corresponds to higher ambient temperatures, and this pattern is clearly observed on both sides of the equator, which also provides guidance for selecting regions suitable for this research deployment and application. However, regions with abundant solar radiation and a need for cooling are not necessarily fully compatible with this research, as this also depends closely on the local water vapor pressure and chemical potential, as illustrated in Figure S66 and S67. Owing to the photothermal process that drives desorption in this research, regions with high annual radiation and high humidity such as Central Africa, South Asia, and South America-exhibit good potential for this research desorption. In contrast, regions with insufficient solar radiation and low humidity, such as Greenland, show negligible performance. On the other hand, the chemical potential of water vapor can be used to evaluate the feasibility of water vapor adsorption and desorption under different environmental conditions. Therefore, to achieve spontaneous adsorption of water vapor from the environment to this research, practical deployment can be guided by local chemical potential values to maximize the device’s potential. For example, in Northwest China and North Africa, the average water vapor chemical potential during the spring and winter and summer and autumn seasons is approximately lower than -240 KJ mol^-1^, whereas in Central Africa and Brazil, the chemical potential during both seasons is approximately -235 KJ mol^-1^. Consequently, this research is suitable for regions such as Central Africa and Brazil, but may not be suitable for Northwest China and North Africa. A comprehensive consideration of global cumulative solar irradiation, ambient temperature, water vapor pressure distributions, and chemical potential distributions is essential for optimizing this research deployment, effectively avoiding performance redundancy and reducing the economic cost of system implementation.

The relevant calculation procedures are described as follows. First, the solar radiation received outdoors is calculated as:

$$\begin{aligned} {\bar{\text{E}}}_{\text{Sun}}\text{=}\int\text{P}_{\text{Sun}}\text{∙}\text{A}\text{∙}\text{dt}\#\left( S5 \right) \end{aligned}$$

where ${\bar{\text{E}}}_{\text{Sun}}$ is the daily solar radiation received by the PV panel, $\text{P}_{\text{Sun}}$ is the solar light intensity, $\text{A}$ is the light-receiving area of the PV panel (0.0324 m^2^), $\text{t}$ is the daily operating duration of the outdoor experiment (9 h), $\text{E}_{\text{Sun}_{\text{1}}}$, $\text{E}_{\text{Sun}_{\text{2}}}$represent the daily solar radiation received by the PV panel on October 13 and October 15, respectively, ${\bar{\text{E}}}_{\text{Sun}}$ is the average cumulative radiation over the two days. After calculating the average daily energy storage, the relationship between light intensity and the power generation pattern of the bifacial PV panel is calculated as:

$$\begin{aligned} {\bar{\text{E}}}_{\text{Storage}}\text{=}\frac{\text{E}_{\text{1}}\text{+}\text{E}_{\text{2}}}{\text{2}}\#\left( S6 \right) \end{aligned}$$

$$\begin{aligned} {\text{E}^{\text{'}}}_{\text{Storage}}\text{=}\frac{{\bar{\text{E}}}_{\text{Storage}}}{{\bar{\text{E}}}_{\text{Sun}}}\#\left( S7 \right) \end{aligned}$$

where ${\bar{\text{E}}}_{\text{Storage}}$ is the average energy storage over 9 h of outdoor operation across two days，$\text{E}_{\text{1}}$、$\text{E}_{\text{2}}$ are the actual energy storage of operating for 9 h on October 13 and October 15, respectively, ${\text{E}^{\text{'}}}_{\text{Storage}}$ is the energy storage per unit solar radiation. The maximum power of the PV panel used in the outdoor experiment is calculated as:

$$\begin{aligned} \text{P}_{\text{max}}\text{=}\text{V}_{\text{oc}}\text{×}\text{I}_{\text{sc}}\text{×}\text{FF}\#\left( S8 \right) \end{aligned}$$

$$\begin{aligned} \text{N}_{\text{PV}}\text{=}\frac{\text{C}_{\text{global}}}{\text{P}_{\text{max}}}\#\left( S9 \right) \end{aligned}$$

$$\begin{aligned} \text{A}_{\text{global}}\text{=}\text{N}_{\text{PV}}\text{×}\text{A}\#\left( S10 \right) \end{aligned}$$

where$\text{ }\text{P}_{\text{max}}$ is the maximum power generated by the outdoors PV panel, $\text{V}_{\text{oc}}$-7.8 V, $\text{I}_{\text{sc}}$-0.6 A, $\text{FF}$ taken as 75%. The calculated $\text{P}_{\text{max}}$ is approximately 3.5 W, corresponding to the rated power of the outdoor experimental PV panel. $\text{N}_{\text{PV}}$ is the equivalent number of PV panels, $\text{C}_{\text{global}}$ is the global installed PV capacity (in 2022, bifacial PV panels accounted for approximately 60% of the global installed capacity),^[9]^ and $\text{A}_{\text{global}}$ is the total area of bifacial PV panels corresponding to the global installed capacity, $\text{A}$ is the front side light-receiving area of a single PV panel. After obtaining the total deployment area of bifacial PV panels, the total received radiation, power generation, and carbon emission reduction are calculated as:

$$\begin{aligned} \text{E}_{\text{abs}}\text{=}\text{A}_{\text{global}}\text{×}\text{H}\#\left( S11 \right) \end{aligned}$$

$$\begin{aligned} \text{e}_{\text{Storage}}\text{=}{\text{E}^{\text{'}}}_{\text{Storage}}\text{×}\text{E}_{\text{abs}}\#\left( S12 \right) \end{aligned}$$

$$\begin{aligned} \text{M}_{\text{CO}_{\text{2}}}\text{=}\text{e}_{\text{Storage}}\text{×}\text{f}_{\text{CO}_{\text{2}}}\#\left( S13 \right) \end{aligned}$$

where $\text{E}_{\text{abs}}$ is the total solar radiation received by the PV panels, $\text{H}$ is the average monthly total radiation received in each countury, $\text{e}_{\text{Storage}}$ is the annual additional power generation of the PV system. $\text{M}_{\text{CO}_{\text{2}}}$ is the annual CO_2_ emission reduction, $\text{f}_{\text{CO}_{\text{2}}}$ is the average carbon emission intensity of each country.^[10]^

**Figure S64.** Global cumulative solar irradiation.

**Figure S65.** Global ambient temperature distributions.

**Figure S66.** Global water vapor pressure distributions.

**Figure S67.** Global chemical potential distributions.

**Figure S68.** Geographical distribution of carbon reduction potential.

**References**

1.	NIST X-ray photoelectron spectroscopy database. https://srdata.nist.gov/xps/EnergyTypeElement

2.	Nansé, G.; Papirer, E.; Fioux, P.; Moguet, F.; Tressaud, A. Fluorination of Carbon Blacks: an X-Ray Photoelectron Spectroscopy Study: I. a Literature Review of XPS Studies of Fluorinated Carbons. XPS Investigation of Some Reference Compounds. *Carbon*. **1997**, *35*, 175-194. https://doi.org/10.1016/S0008-6223(96)00095-4.

3.	Spanring, J.; Buchgraber, C.; Ebel, M. F.; Svagera, R.; Kern, W. UV Assisted Surface Modification of Polystyrene in the Presence of Trialkylsilanes. *Macromol. Chem. Phys*. **2005**, *206*, 2248-2256. https://doi.org/10.1002/macp.200500304.

4.	Wang, H.; Wu, P.; Wu, Z.; Shi, L.; Cheng, L. New Insight into the Electronic Structure of SiF_4_: Synergistic Back-Donation and the Eighteen-Electron Rule. *Phys. Chem. Chem. Phys*. **2022**, *24*, 17679-17685. https://doi.org/10.1039/D2CP01607A.

5.	Kresse, G.; Furthmüller, J. Efficiency of Ab-Initio Total Energy Calculations for Metals and Semiconductors Using a Plane-Wave Basis Set. *Comput. Mater. Sci*. **1996**, *6*, 15-50. https://doi.org/10.1016/0927-0256(96)00008-0.

6.	Perdew, J. P.; Burke, K.; Ernzerhof, M. Generalized Gradient Approximation Made Simple. *Phys. Rev. Lett*. **1996**, *77*, 3865-3868. https://doi.org/10.1103/PhysRevLett.77.3865.

7.	Blöchl, P. E. Projector Augmented-Wave Method. *Phys. Rev. B*. **1994**, *50*, 17953-17979. https://doi.org/10.1103/PhysRevB.50.17953.

8.	Grimme, S. Semiempirical GGA‐Type Density Functional Constructed with a Long‐Range Dispersion Correction. *J. Comput. Chem*. **2006**, *27*, 1787-1799. https://doi.org/10.1002/jcc.20495.

9.	IEA-PVPS. *Trends in Photovoltaic Applications 2022*. (IEA-PVPS, 2022).

10.	Ember. Lifecycle Carbon Intensity of Electricity, 2025. *Our World in Data*. **2026**. https://ourworldindata.org/grapher/carbon-intensity-electricity
